# Development of an antibody fused with an antimicrobial peptide targeting *Pseudomonas aeruginosa*: A new approach to prevent and treat bacterial infections

**DOI:** 10.1371/journal.ppat.1011612

**Published:** 2023-09-07

**Authors:** Kenneth Johnson, James C. Delaney, Thomas Guillard, Fany Reffuveille, Jennifer Varin-Simon, Kai Li, Andrew Wollacott, Eric Frapy, Surin Mong, Hamid Tissire, Karthik Viswanathan, Faycal Touti, Gregory J. Babcock, Zachary Shriver, Bradley L. Pentelute, Obadiah Plante, David Skurnik

**Affiliations:** 1 Visterra, Inc., Waltham, Massachusetts, United States of America; 2 Inserm UMR-S 1250 P3 Cell, Université de Reims-Champagne-Ardenne, Reims, France; 3 CNRS, INSERM, Institut Necker Enfants Malades-INEM, F-75015 Paris, France; Faculté de Médecine, University of Paris City, Paris, France; 4 Department of Chemistry, Massachusetts Institute of Technology, Cambridge, Massachusetts, United States of America; 5 Department of Clinical Microbiology, Fédération Hospitalo-Universitaire Prématurité (FHU PREMA), Necker-Enfants Malades University Hospital, Assistance Publique-Hôpitaux de Paris, University of Paris City, Paris, France; 6 Division of Infectious Diseases, Department of Medicine, Brigham and Women’s Hospital, Harvard Medical School, Boston, Massachusetts, United States of America; University of North Carolina at Chapel Hil, UNITED STATES

## Abstract

The increase in emerging drug resistant Gram-negative bacterial infections is a global concern. In addition, there is growing recognition that compromising the microbiota through the use of broad-spectrum antibiotics can impact long term patient outcomes. Therefore, there is the need to develop new bactericidal strategies to combat Gram-negative infections that would address these specific issues. In this study, we report and characterize one such approach, an antibody-drug conjugate (ADC) that combines (i) targeting the surface of a specific pathogenic organism through a monoclonal antibody with (ii) the high killing activity of an antimicrobial peptide. We focused on a major pathogenic Gram-negative bacterium associated with antibacterial resistance: *Pseudomonas aeruginosa*. To target this organism, we designed an ADC by fusing an antimicrobial peptide to the C-terminal end of the V_H_ and/or V_L_-chain of a monoclonal antibody, VSX, that targets the core of *P*. *aeruginosa* lipopolysaccharide. This ADC demonstrates appropriately minimal levels of toxicity against mammalian cells, rapidly kills *P*. *aeruginosa* strains, and protects mice from *P*. *aeruginosa* lung infection when administered therapeutically. Furthermore, we found that the ADC was synergistic with several classes of antibiotics. This approach described in this study might result in a broadly useful strategy for targeting specific pathogenic microorganisms without further augmenting antibiotic resistance.

## Author summary

The increasing incidence of emerging drug resistant bacterial infections is a worldwide public health issue. Infections caused by antibiotic-resistant Gram-negative pathogens are particularly concerning. In addition, there is now growing recognition that disruption of the microbiota through the use of broad-spectrum antibiotics can have detrimental effects on long-term patient outcomes. Therefore, there is a need to develop new bactericidal strategies to combat Gram-negative infections while preserving the microbiota and avoiding the enhancement of antibiotic resistance. Here, we report on and characterize one such approach by using a specific monoclonal antibody associated with the potent killing activity of antimicrobial peptides in the form of an antibody-drug conjugate (ADC). The selected pathogenic bacterium was *Pseudomonas aeruginosa*, which presents numerous markers of both innate and acquired antibiotic resistance. The ADC lacked significant cytotoxicity against mammalian cells and was shown to be effective both *in vitro* and *in vivo* against *P*. *aeruginosa*.

## Introduction

Antimicrobial resistance is a serious and growing public health threat [1]. The Centers for Disease Control and Prevention (CDC) estimates that more than 2.6 million people in the United States are infected each year with antibiotic-resistant microorganisms, with at least 44,000 dying as a result [2]. Of the various resistant human pathogens, Gram-negative bacteria–particularly the carbapenem-resistant *Enterobacterales* (CRE), the multi-drug resistant (MDR) *Pseudomonas aeruginosa* and *Acinetobacter baumannii–*are among the most concerning [3, 4]. For example, *P*. *aeruginosa*’s intrinsic resistance to many antibiotics limits treatment options. Furthermore, the acquisition of resistance elements leading to MDR and even pan-resistant strains has created a public health concern with potentially untreatable *P*. *aeruginosa* strains [5]. The CDC’s 2019 report designated MDR *P*. *aeruginosa* as a “Serious Threat” [2], and the World Health Organization classified carbapenem-resistant *P*. *aeruginosa* as one of two “Priority 1: Critical Threats” in 2017. In addition, carbapenem-resistant *P*. *aeruginosa* strains were recently reported to be more fit and virulent *in vivo* [6, 7]. This emerging situation warrants the urgent development of new types of treatments and/or approaches for either preventing or treating *P*. *aeruginosa* infections [8].

Several classes of antibiotics are able to elicit rapid bactericidal effects, with a >99.9% reduction in the bacterial counts within four hours at peak concentrations [9]. However, this very high killing ability is associated with several shortcomings. Firstly, these treatments induce strong selective pressure, such that their use invariably leads to the rapid emergence and dissemination of antibiotic resistance [10–12]. Secondly, broad-spectrum antibiotics act not only on the pathogenic strains but also target the host microbiota, altering quickly and sometimes persistently its taxonomic, genomic and functional capacities and leading to potential negative consequences for the patient [13, 14]. Lastly, it has also been suggested that compared with antimicrobial agents alone, antibody conjugates are less toxic [15].

Thus, there is a need to develop novel targeted strategies to treat pathogenic organisms (particularly Gram-negative pathogens) with high killing abilities but also with as few of these limitations as possible.

To this end, we describe the development and characterization of a new strategy to treat bacterial infections–even those caused by MDR or pan-resistant strains–by combining the unique specificity of a monoclonal antibody (mAb, referred hereafter to as VSX [16]) with the direct-acting antibacterial activity of an antimicrobial peptide (AMP). By directly linking the AMP to VSX, we created an antibody-drug conjugate (ADC). While the principle of employing an antibody conjugate has recently been described in the treatment of bacterial infections, these first reports mainly employed antibody-antibiotic conjugates [17]. Thus, in this work, and for the first time to the best of our knowledge, we present an antibody conjugated to an AMP enabling direct bactericidal activity against Gram-negative bacteria by targeting the organism’s outer membrane. Our approach therefore combines adding a strong antimicrobial activity with the benefits of a specific approach associated with the use of an antibody. To exemplify this approach, we focused on *P*. *aeruginosa* [18].

We find that our VSX-AMP constructs (henceforth referred to as ADCs) function with both direct bactericidal activity and an effector function through the Fc domain of the antibody. We show here that this construct shows potent, selective activity *in vitro* and *in vivo*, with specific *P*. *aeruginosa* killing activity and little to no detectable non-specific cytotoxicity against mammalian cells. Additionally, ADC constructs based on VSX and an AMP have a direct action at the outer membrane surface, which does not require internalization of the ADC and thereby circumvents the need for an agent that must pass through the double membrane of Gram-negative pathogens. Taken together, the data presented here demonstrate that our ADC constructs provide a therapeutic option for managing *P*. *aeruginosa* infections, promoting antibiotic stewardship, and sparing the host microbiota.

## Results and discussion

### Selection of an antibody

**Fig 1:**
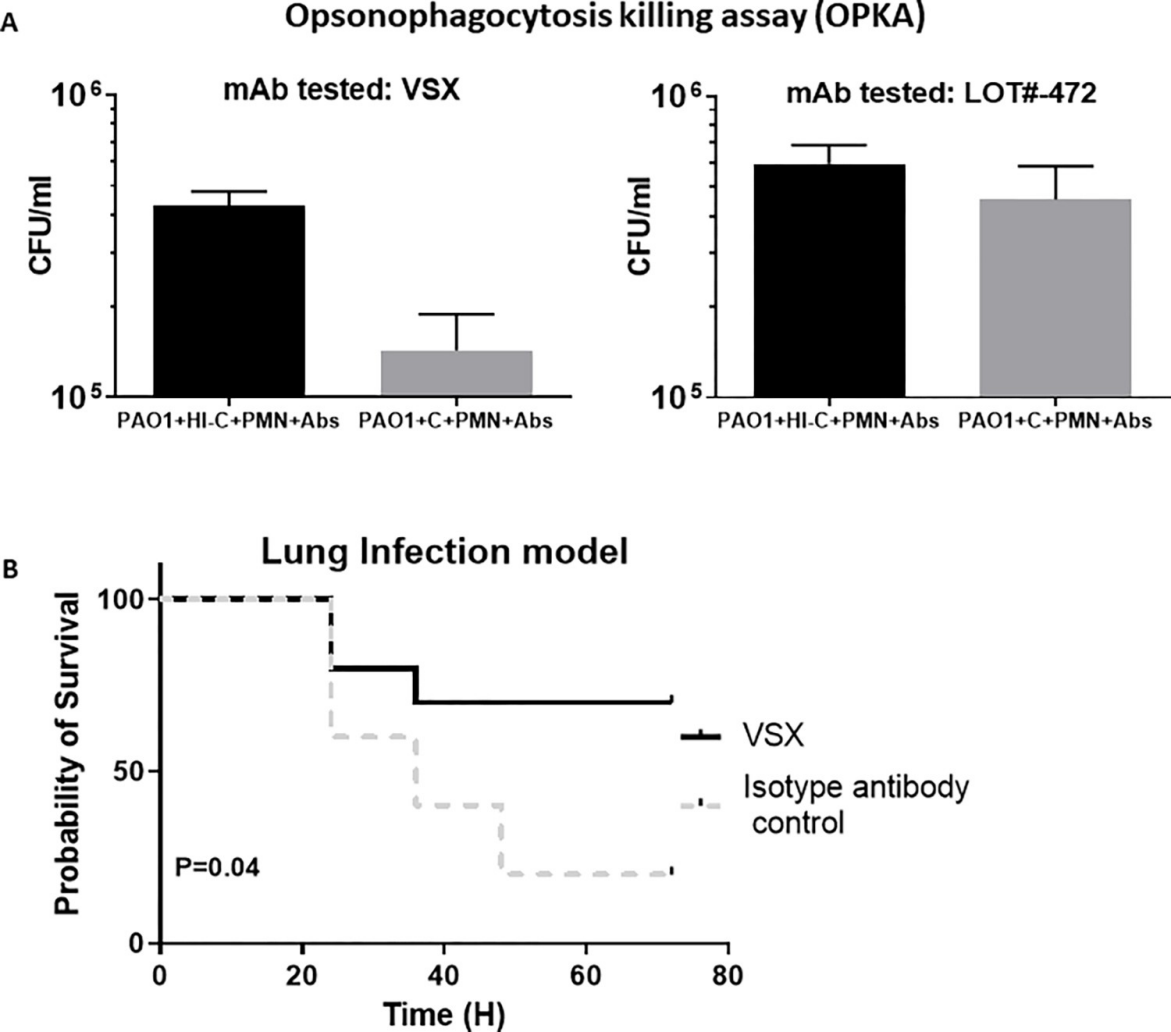
*In vitro* and *in vivo* activities of the monoclonal antibody (mAb) VSX targeting *P*. *aeruginosa* core LPS. **(A)** *In vitro* killing of *P*. *aeruginosa* PAO1 in the presence of PMN and complement (OPKA) by VSX. The lot#-472 is representative of mAbs able to bind *P*. *aeruginosa* but without any detectable OPKA activity. C = complement. HIC = heat inactivated complement. PMN = polymorphonuclear leukocytes. Abs = antibodies. **(B)** *Acute lung infection model*. Challenge dose: 2x10^6^ CFU. Inoculation: intranasal, 10^6^ CFUs in each nostril. Mice = 10 per group (two experiments with 5 animals per group each time). mAbs were injected IP 4 hours post-infection. Dose of the mAbs: VSX = 15 mg/kg. Control mAb (against *Clostridioides difficile*) = 15 mg/kg. P-value = 0.04, measured in a log-rank test.

The first step in the construction of an ADC targeting *P*. *aeruginosa* is to identify an antibody that binds with high affinity/avidity to the outer membrane and targets a conserved epitope. VSX, as described previously [16], is such an antibody. It has been shown to engage monosaccharides within the inner core of LPS; these include phosphorylated heptose, which is conserved across *Pseudomonas* species (including *P*. *aeruginosa*). By engaging such a highly conserved site on a predominant antigen (with approximately one million copies at the outer membrane [19]), VSX has the potential to be the basis of a broad-spectrum immunotherapy for *P*. *aeruginosa* infection. Indeed, VSX has been shown to bind to a panel of 100 different isolates of *P*. *aeruginosa* (including both rough and smooth variants); quantitative binding to 10 of these strains yielded dissociation constants of <1 nM (Figs 1A, in ref. 6 and S3). Taken together, these data suggested to us that VSX could form the basis of an ADC.

As a first step in the process of manufacturing an ADC, we characterized the biological activity of the VSX antibody itself, to establish a baseline for additional studies. In particular, we examined the ability for VSX alone to kill *P*. *aeruginosa* in the presence of polymorphonuclear leukocytes (PMNs) and complement. VSX demonstrated activity *in vitro* using the opsonophagocyotsis killing assay (OPKA, **Fig 1A**) against the reference strain PAO1 previously used in OPKA assays [20]. *In vivo*, we used a mouse model of pneumonia that we have previously reported on [6]. As shown in **Fig 1B**, VSX injected intraperitoneally four hours post-infection was able to significantly protect mice infected by *P*. *aeruginosa* in our acute lung infection model (P = 0.04, Log-Rank test). Notably, for this analysis, we used a single, relatively high dose level of 15 mg/kg, which is consistent with the dose levels of between 5–20 mg/kg selected for other antimicrobial antibodies [21]. Therefore, these data suggest that VSX is a suitable basis for construction of an ADC. However, the activity of VSX alone is likely insufficient for several reasons because notably (i) the antibody itself does not appear to interfere with a vital function of the organism and thus is not directly bactericidal; and (ii) it requires engagement with the host’s immune system; in a situation in which a patient is immunocompromised, the activity of VSX might be much reduced.

### Selection of an AMP for conjugation with VSX

To improve the *in vitro* and *in vivo* killing activity of VSX (**Fig 1**), we sought to arm the antibody with direct killing activity, without the need for recruitment of either complement or PMNs [22]. Since VSX targets the outer membrane surface of *P*. *aeruginosa*, we sought to enable direct bactericidal activity through the addition of an AMP—many of which have demonstrated the ability to act on the outer membrane [23]. In addition, we chose to focus on AMPs due to their rapid killing activity and the proven efficacy of their mechanism of action, which approximated that of the last line of defense of our current antibiotics (the polymyxins) [24].

**Fig 2:**
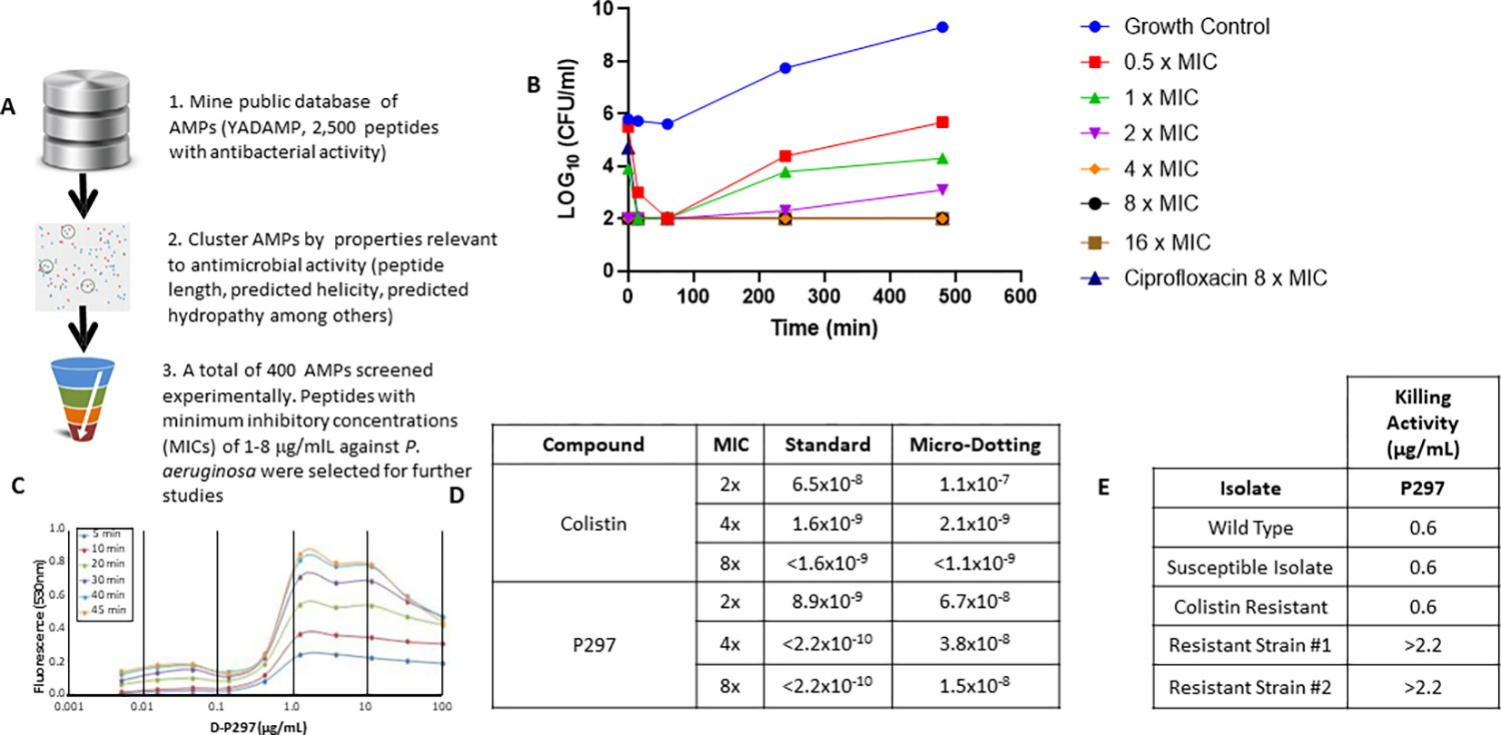
*Design and screening of AMPs*. (**A**) The workflow for identification of an AMP to deploy in the construction of an ADC. (**B**) The AMP P297 showed rapid bactericidal activity in a time-kill assay using *P*. *aeruginosa* ATCC 27853. A growth control was compared with P297 at 0.5, 1, 2, 4, 8, or 16x MIC of peptide or with ciprofloxacin at 8x MIC. Note that the 4x, 8x, 16x, and ciprofloxacin 8x MICs all overlap. (**C**) *Calcein leakage*. The mechanism of action for P297 likely involves membrane disruption, as assessed by measuring calcein leakage from DOPE/DOPG liposomes. Various concentrations of peptide (from 0.05–100 μg/ml) were incubated with liposomes for defined lengths of time (5–45 min). Release of calcein was assessed by measuring the increase in fluorescence at 530 nm, compared with a non-peptide reference. Peptide concentrations above 1 μg/ml resulted in a measurable increase in fluorescence. (**D**) Assessment of resistance rates for P297 vs. colistin, using two different procedures. (**E**) Comparison of wild-type *P*. *aeruginosa* ATCC strain 27853 with two P297-resistant mutants indicates differences in drug sensitivity and phenotype. The mutant strains were less sensitive to P297.

To identify an optimal AMP for use in our ADC, we completed a robust structure-activity campaign to identify AMPs that (i) are active at the cell surface and hence do not require internalization; (ii) are bactericidal against *P*. *aeruginosa*, and (iii) have low hemolytic and cytotoxic activities. To this end, we used a bioinformatics-driven workflow coupled with experimental testing to identify potent AMPs with a high therapeutic index. We implemented a screening strategy that clustered peptides based on their underlying physicochemical properties, followed by characterization of representative members of each cluster. Here, we used YADAMP (http://www.yadamp.unisa.it/), a database of AMPs that contains over 2,500 sequences of peptides with reported antibacterial activity **(Fig 2A, top)**. Next, clustering was performed by utilizing a K-means algorithm and calculated properties that are reportedly relevant to antimicrobial activity (peptide length, predicted helicity, predicted hydropathy, percentage of select amino acids [Lys, Arg, Trp, Cys, and His], and charge at pH 7, pH 5, and pH 9) (**Fig 2A, middle**). There are at least four distinct classes of AMP, based on their secondary structures: β-sheet, α-helix, extended, and loop [25]. Since we were focusing on AMP activity in the context of covalent linkage to an antibody, we eliminated the loop class because of the requirement for disulfides to stabilize the secondary structure. In addition, we eliminated peptides that require oligomerization to elicit cell killing activity. With these limitations in mind, the class of amphipathic α-helical peptides that act at the cell surface clearly represent a desirable peptide class for formation of an ADC. Furthermore, reports indicate that binding of approximately one to ten million AMPs per cell will induce cell killing [26]. Thus, we reasoned that leveraging a multiple drug-to-antibody ratio (DAR) ratio and using the VSX antibody to anchor the AMP in constant close proximity to the *P*. *aeruginosa* outer membrane might create a surface concentration of peptide sufficient to elicit membrane disruption and cell killing.

An initial set of 100 peptides was selected for experimental characterization in a panel of *in vitro* assays involving minimum inhibitory concentration (MIC) testing against multiple bacterial strains, hemolytic evaluation, and cytotoxicity. Once potent peptides with favorable characteristics had been identified, the remaining members of the corresponding cluster were selected for additional experimental characterization. Using this approach, we were able to dramatically reduce the number of peptides that required screening by approximately 6-fold. Over the course of the campaign, ~400 peptides were screened experimentally (**Fig 2A, bottom**).

### Identification of AMP P297

The initial screen identified several α-helical AMPs that possessed MICs of 1–8 μg/ml against two ATCC strains of *P*. *aeruginosa*: *P*. *aeruginosa* ATCC 27853 and *P*. *aeruginosa* ATCC 39324. Some AMPs also had broader activity against other bacterial species (**Table 1**). However, we noted that several of these initial AMPs also demonstrated lytic activity against human red blood cells. One notable exception to this trend was the α-helical sub-class of the cathelicidin family of AMPs. Cathelicidins are a family of structurally diverse AMPs that exert potent antibacterial activity and act as multifunctional effector molecules of innate immunity [27, 28]. Results from multiple sequence variants from this family are outlined in **Table 1.** In particular, one member of this class (cathelicidin-BF [29], highlighted by one derivative: P297) demonstrated potent MIC values against the *P*. *aeruginosa* ATCC strains, low hemolytic activity, and selectivity that resulted in much less killing of mammalian cells. Time-course experiments with P297 indicated that at concentrations of four times the MIC or greater, the peptide was able to rapidly reduce *P*. *aeruginosa* titers greater than 1000-fold—confirming that the peptide was bactericidal and not merely bacteriostatic (**Fig 2B**). Consistently with this rapid onset of action, activity for P297 was higher in a killing assay (see *Materials and Methods*) than in a more traditional MIC assay (EC_50_ of 0.14–0.28 μg/ml in the killing assay, compared with 2–4 μg/ml in an MIC assay). The killing ability of P297 was confirmed and extended upon examination of the peptide’s activity towards the third-generation cephalosporin-resistant strains BAA-2110 and BAA-2114, where the peptide exhibited a similar EC_50_ (0.14–0.28 μg/ml).

**Table 1:** *In vitro* Activity and Toxicity of Representative Peptide Variants.

		MIC (μg/ml)				Hemolysis		Human Serum	Cytotox
Sample	Sequence	*P*. *aeruginosa* ATCC 27853	*P*. *aeruginosa* ATCC 39324	*E*. *coli* ATCC 25922	*S*. *aureus* 29213	MLC/ MIC	PLC/ MIC	hsMIC/MIC	CC_50_ (μg/ml)
P261	GGGGIGKFLKKAKKFGKAFVKILKK	16	8	4	32	> 8	> 8	2	250
P265	GGGLLGDFFRKSKEKIGKEFKRIVQRIKDFLRNLVPRTES	32	16	32	> 128	> 4	1	> 4	90
P267	GGGGRFKRFRKKFKKLFKKLSPVIPLLHLG	4	4	4	16	> 32	> 32	0.5	70
P271	GGGGLRKRLRKFRNKIKEKLKKIGQKIQGLLPKLA	16	8	4	16	> 8	4	2	250
P292	GGGHTASDAAAAAALTAANAAAAAAASMA	> 128	> 128	> 128	> 128	ND	< 1	ND	250
P293	GGGGLRRLGRKIAHGIKKYGPTILRIIRIAG	8	2	4	4	16	4	4	70
P294	GGGRGLRRLGRKIAHGVKKYGPTVLRIIKKYG	64	2	4	4	> 2	> 2	1	120
P295	GGGGRFKRFRKKFKKLFKKLSPVIPLLHLG	4	4	4	16	> 32	> 32	1	95
P296	GGGKRFKKFFKKLKNSVKKRAKKFFKKPRVIGVSIPF	4	2	4	16	> 32	32	NA	400
P297	GGGKFFRKLKKSVKKRAKEFFKKPRVIGVSIPF	4 (1.0)	2 (0.5)	4 (1.0)	32 (8.3)	> 32	> 32	2	780
Ctl.	Ceftazadime	1 (1.8)	1 (1.8)	0.25 (0.5)	4 (7.3)	16	8	1	550

MIC, minimum inhibitory concentration, the numbers in parentheses are molar equivalents; MLC, mean lytic concentration; PLC, partial lytic concentration; hsMIC, MIC in human serum; CC_50_: the concentration at which 50% cytotoxicity of mammalian cells is observed; ND, not determined; NA, no activity in human serum.

### Characterization of AMP P297

Given its activity profile, we sought to confirm the overall structure of P297 and ensure that it was concordant with the peptide’s putative mechanism of action. To this end, we first employed circular dichroism to analyze the peptide’s secondary structure. Inspection of P297’s spectrum indicated that in aqueous solution, P297 did not adopt an appreciable secondary structure, with the minimum at approximately 198 nm corresponding to the π-π* transition of a random coil (**S1 Fig**). However, in the presence of 40% 2,2,2-trifluoroethanol (a nonpolar solvent that has been used to promote native-like α-helical structures in peptides with intrinsic α-helix forming properties and represents, to a certain extent, the hydrophobic environment of the lipid membrane), a strong peak at 196 nm was indicative of α-helix formation.

Additional mechanistic studies confirmed that P297 likely works through membrane disruption (**Fig 2C,** calcein leakage). In this case, model membranes were created with DOPE/DOPG liposomes and loaded with calcein as a reporter [30]. When liposomes were subjected to P297, rapid disruption of the lipid layer resulted in release of the dye (as measured by fluorescence). This release was P297 concentration-dependent; furthermore, time-course studies indicated that membrane disruption was rapid and reached completion in less than 45 minutes.

In addition to the above structure-activity studies, we sought to understand the impact of peptide P297 administration on *P*. *aeruginosa* and, in particular, the organism’s ability to develop resistance to P297. Resistance was assessed with two methods (see *Methods*). For both methods, we compared the frequency of emergent resistance to P297 to that of polymyxin B (colistin). We noted similar or lower frequency of mutations (on the order of 10^−8^ to 10^−10^) for P297, compared with polymyxin B (**Fig 2D**). During our resistant rate determination assays with P297, we isolated a mutant strain that demonstrated significant resistance in killing assays and MIC determinations (**Fig 2E**).

Mutations that confer antibiotic resistance often involve modifications of the bacteria, which can lead to sub-optimal biological functioning. To determine whether there was a fitness cost associated with acquisition of resistance to P297, a competitive fitness assay was run on the wild-type *P*. *aeruginosa* ATCC 27853 vs. its mutant resistant strain. Briefly, we mixed 10^6^, 10^5^, and 10^4^ of each strain. At 24 hours, we plated 50 μl of serial dilutions of the mixed cultures onto blood agar plates (BAPs) to determine the CFU/ml (see the Methods section for more details). We found that the relative fitness value for the resistant strains was 0.855; this compared favorably with values reported for strains resistant to the majority of antibiotics, where a significant fitness cost has been associated with a mean fitness value of 0.88 [31].

Lastly, we interpreted the mechanistic and resistance studies in the context of the peptide’s specificity, which is critical for the ultimate ADC construct. To this end, P297 exhibited a relatively high specificity (therapeutic index) in our *in vitro* assays. One key metric that we employed and that has demonstrated to be a sensitive assay of toxicity is red blood cell (RBC) hemolysis [32]. For this assessment, we employed both the mean lytic concentration (MLC, i.e. the concentration of peptide that induced 100% hemolysis) and the minimal concentration at which red blood cell hemolysis is first observed (defined as the partial lytic concentration, PLC). With both measurements, the levels of P297 at which RBC hemolysis was observed were 10-fold greater than the MIC. Furthermore, the cytotoxicity of P297 against the representative mammalian cell line 293T was approximately 200-fold greater than the MIC. Taken together, these results confirm (i) that P297 is an α-helical, bactericidal peptide that is able to rapidly kill *P*. *aeruginosa*, (ii) that it has a relatively high barrier to resistance development, (iii) that resistance could occur through selection of spontaneous mutants, (iv) that a fitness cost was associated with acquisition of this resistance to the AMP, and (v) that P297 had a high therapeutic index. Therefore, P297 was selected for further peptide design.

### Improvement of P297 and selection of D297

Further analysis of P297 indicated that although it was highly active and specific *in vitro*, its activity decreased substantially over time in the presence of human or mouse serum (**S2 Fig**). LC-MS analysis of P297 in the presence of serum demonstrated that the peptide was likely adsorbing to serum components—presumably proteins—and was also being cleaved by serum proteases; its measured half-life was on the order of 20 minutes. Therefore, we set out to modify the properties of P297 to make it more amenable to long exposure times *in vivo*, especially given that antibody levels can be sustained for days. As a first modification, we replaced the l-amino acids of P297 (which adopts a right-handed helix) with d-amino acids, resulting in a new peptide referred to as D297. Notably, substitution of l-amino acids with d-amino acids has been shown previously to decrease protease sensitivity and increase the serum stability of peptides [33, 34]. Circular dichroism analysis of D297 confirmed that it adopted a left-handed helix in the presence of 2,2,2-trifluoroethanol. *In vitro* analysis of D297 indicated that it possessed equivalent potency against *P*. *aeruginosa* when compared with P297, with the added benefit of stability in serum (**S2 Fig**). As with P297, D297 had low hemolytic and cytotoxic activity against RBCs and mammalian cells, respectively, demonstrating specificity for bacterial membranes. Thus, by converting to the D-peptide sequence, we were able to retain bactericidal activity, enhance serum stability, and preserve the AMP’s lack of hemolytic and cytotoxicity. Lastly, we confirmed that the D297 peptide had activity against an MDR strain of *P*. *aeruginosa*. For this assessment, *P*. *aeruginosa* strain ATCC (BAA-) 2108 was selected; this strain has resistance to most carbapenems and cephalosporins and intermediate resistance to third-generation fluoroquinolones. Despite the organism’s resistance profile, D297 demonstrated significant activity against it in the MIC assay (4 μg/ml).

### Construction of VSX conjugates

**Fig 3:**
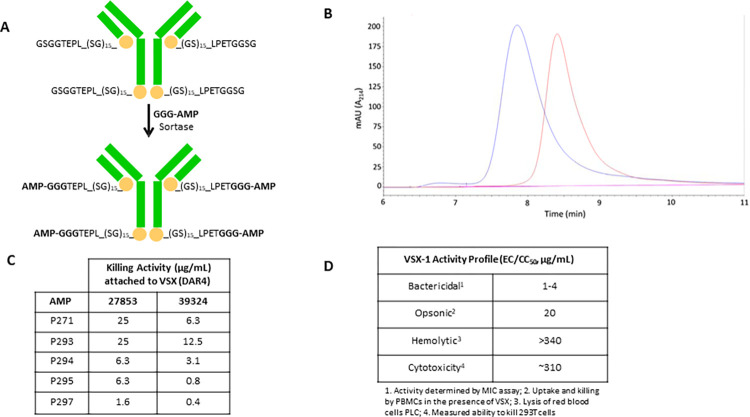
*Synthesis*, *characterization and* in vitro *evaluation of VSX conjugates*. (**A**) Synthesis of antibody-AMP conjugates, using sortase ligation. VSX was expressed in Expi293 cells with a (GS)_15_ flexible linker and a sortase acceptor tag (LPETGGSG) present at the C-terminus of both the light chain and the heavy chain. Next, a (GGG)-modified AMP (P297, for example) was added covalently via incubation with recombinantly produced sortase for a target DAR of 4. This linker/sortase addition strategy was employed for all constructs analyzed in the present study. (**B**) Size exclusion chromatography (SEC-HPLC) of VSX (red line) and VSX conjugate (blue line), indicating an earlier shift in elution time for the modified construct vs. the starting antibody. The constructs did not differ significantly in the peak width and height, indicating a relative degree of homogeneity in the sortase modification. (**C**) *In vitro* killing activity of VSX conjugates. *P*. *aeruginosa* ATCC strains 27853 or 39324 were treated with VSX conjugates with a DAR of ~4 containing AMP peptides 271, 293, 294, 295, or 297. Conjugate IgG concentrations that resulted in 50% killing are recorded. (**D**) VSX-1 (VSX with peptide P297 and a DAR of ~4) was characterized in several assays, including *in vitro* bactericidal activity, opsonic activity, hemolytic activity, and cytotoxic activity.

As mentioned earlier, the VSX antibody alone has several shortcomings, such that it is unlikely–by itself–to be a strong bactericidal agent. Even the D297 engineered peptide alone is anticipated to have a number of shortcomings. While D297 seems to be a strong bactericidal agent with rapid onset of action and high barrier to resistance, (i) it still has some residual activity against mammalian cells, and (ii) its half-life (though long) can still be improved. Therefore, having selected VSX as the antibody scaffold and D297 as the AMP, we produced and tested multiple ADC constructs of VSX-D297. Since both the peptide and the antibody could, in theory, be produced recombinantly, our initial efforts focused on their expression within a genetic cassette in CHO cells. Our efforts were unsuccessful, resulting in poor cell viability and low levels of intact construct. Upon selection of D297 as the lead peptide (containing D-amino acids, which are incompatible with biosynthetic incorporation), we elected instead to use an enzymatic ligation strategy employing sortase A (SrtA, **Fig 3A**). The SrtA method [35] involved recombinantly expressing VSX with a SrtA-ligatable tag and then enzymatically coupling the tagged VSX with the chemically synthesized D297 [35]. ADCs were produced as C-terminal variants of either the heavy chain (HC), or light chain (LC) or both chains (dual). All constructs employed a glycine-serine repeat peptide linker containing the LPETGGSG sortase site. Conjugates with HC or LC attachment had a DAR of ~2 (greater than 1.8, as assessed experimentally by mass spectrometry) and those with dual conjugate had a DAR of ~4 (greater than 3.6, as assessed experimentally by mass spectrometry) (**S3 Fig**). For quality control purposes, our ADCs were all analyzed by mass spectrometry (LC-MS), SDS-PAGE and SE-HPLC. The couplings were efficient, routinely converting at >90% per site, as determined by LC-MS. D297 was attached in all cases with a 30-amino-acid (GS)_15_- linker, to maintain flexibility between the terminal HC/LC residue and the sortase signal sequence. As a consequence of this flexibility, SE-HPLC analysis showed a homogenous product with no signs of aggregation (**Fig 3B**). We considered this to be important because aggregation is a common concern with ADCs.

### In vitro killing and the therapeutic index of the ADC VSX-1

Using the VSX antibody, a series of ADC constructs was evaluated *in vitro* for direct bactericidal activity against *P*. *aeruginosa* (**Fig 3C**). From these initial constructs, we were able to conclude that (i) the relative order of activity for the conjugation sites was (HC + LC) > HC > LC and (ii) consistently with its activity as a peptide, the P297 constructs yielded the greatest killing. Based on these data, we constructed an ADC (hereafter referred to as VSX-1) that contained the VSX antibody and a DAR of 4 with the peptide D297 ligated to the C-terminus of each HC and LC (HC+LC) and employing the (GS)_15_ linker (**Fig 3B)**. As observed with D297 alone, VSX-1 had a high therapeutic index, minimal hemolytic activity, and negligible cytotoxic activity against 293T cells (**Fig 3D**). Lastly, killing activity was observed across four *P*. *aeruginosa* strains, including the resistant strains BAA-2110 and BAA-2114.

To confirm the ADC’s specific killing of *P*. *aeruginosa* compared with the lack of specific killing by the peptide alone, the activities of the antibody, the peptide, and VSX-1 were assessed in a mixed culture of *P*. *aeruginosa*, *E*. *coli*, and *K*. *pneumoniae* (**S4 Fig**). Notably, the peptide alone (**S4 Fig, left panel**) showed potent but non-selective killing, with activity detected against both *P*. *aeruginosa* and *E*. *coli*. Consistently with its requirement for the presence of complement or PMNs for detectable activity, the VSX (antibody) alone exhibited no direct killing in their absence (**S4 Fig, middle panel**). Lastly, the ADC combination specifically killed *P*. *aeruginosa* and had no effect on *E*. *coli*, confirming the high specificity of the ADC compared with the AMP alone. (**S4 Fig, right panel**).

### Other in vitro properties of VSX-1

**Fig 4:**
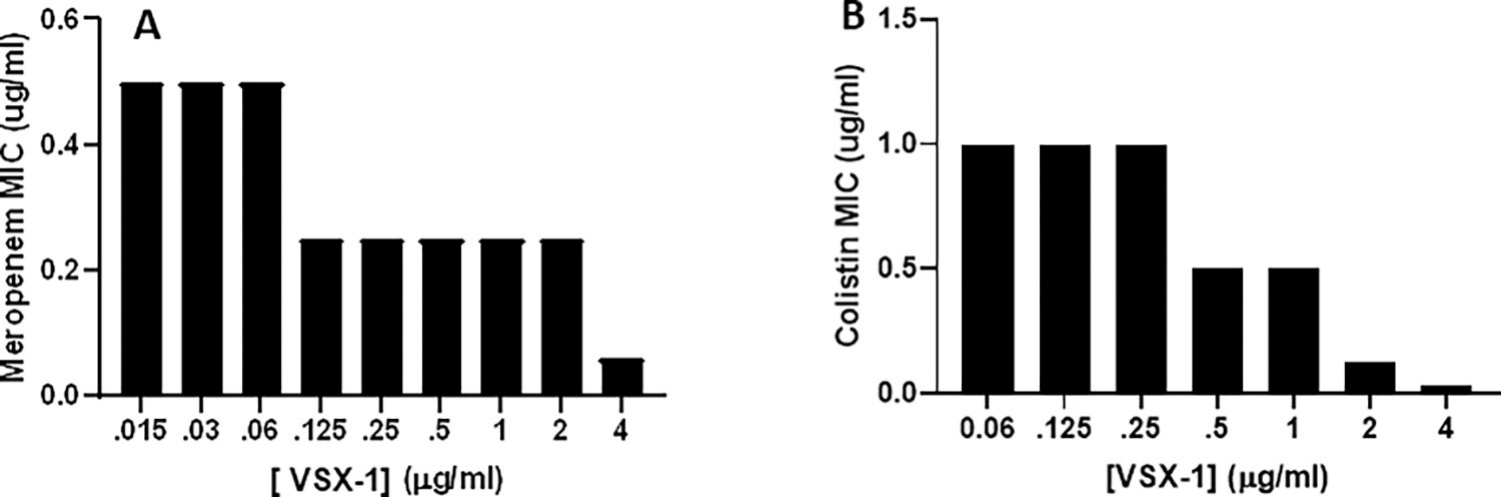
Synergy of P297 with various antibiotic classes. MICs for both meropenem and colistin were determined alone and then in the presence of varying concentrations of P297, in order to measure the effect on the MIC for P. aeruginosa ATCC 27853. (**A**) Examination of the MIC for meropenem in the presence of various concentrations of P297. (**B**) As (**A**), except that colistin was used as the antibiotic. Not all antibiotics demonstrated synergy; for example, the aminoglycoside tobramycin did not exhibit an enhanced MIC in the presence of P297.

(i) *The OPKA (opsonophagocytic killing assay)*. The ADC’s killing properties were also evaluated in an OPKA, to confirm the intactness of the antibody. The ADC was highly active at 10 μg/ml, resulting in a 10-fold bacterial reduction in the presence of heat-inactivated complement and greater than 1000-fold killing activity with non-heat inactivated complement, in the presence of polymorphonuclear neutrophils. As in the OPKA of VSX, the *P*. *aeruginosa* PAO1 strain was used [20]. (ii) *Synergy with antibiotics*. Given that VSX-1 acts at the outer membrane of *P*. *aeruginosa*, we reasoned that it might demonstrate synergy with existing antibiotics that cross the double membrane and reach intracellular targets. Indeed, we found that VSX-1 appeared to increase the potency of conventional anti-*Pseudomonas aeruginosa* antibiotics (*e*.*g*. carbapenems and polymyxin B), with a 10-fold or more reduction in the MIC (**Fig 4**). Indeed, we found that increasing amounts of VSX-1 (from 0.015 to 4 μg/mL) lowered the observed MIC for meropenem from 0.5 to <0.1 μg/ml and that for colistin from ~1 μg/ml to <0.1 μg/ml (**Fig 4**). This result is likely caused by the AMP increasing the outer membrane permeability. (iii) ***LPS-mediated TLR4 activation inhibition***. Lastly, other additional mechanisms of protection that could be mediated by VSX were also explored. Specifically, it has been reported that shedding of LPS can cause pathophysiological manifestations upon hyperstimulation of the host immune system–initiated by activation of toll-like receptors (TLRs), which can then lead to septic shock [36]. Given this pathophysiology, an anti-LPS antibody might play a vital role in neutralizing LPS by limiting LPS shedding, promoting its serum clearance, and inhibiting the LPS-mediated binding to TLR and concomitant activation of the immune system. Furthermore, a HEK-blue reporter assay of the activation of TLR4 receptor by free LPS demonstrated that VSX inhibits LPS-mediated TLR4 activation (**S5 Fig**).

### In vivo activity of VSX-1 in animal models of infections caused by P. aeruginosa strains

To investigate the *in vivo* activity of VSX-1, we focused on lung infection models for several reasons. Primarily, the lung is a common site for *P*. *aeruginosa* infection, which leads to community/hospital-acquired pneumonia and negative outcomes, with mortality rates of up to 30–60% in some studies [37]. As such, it presents one area of likely clinical use for a pathogen-specific ADC.

**Fig 5:**
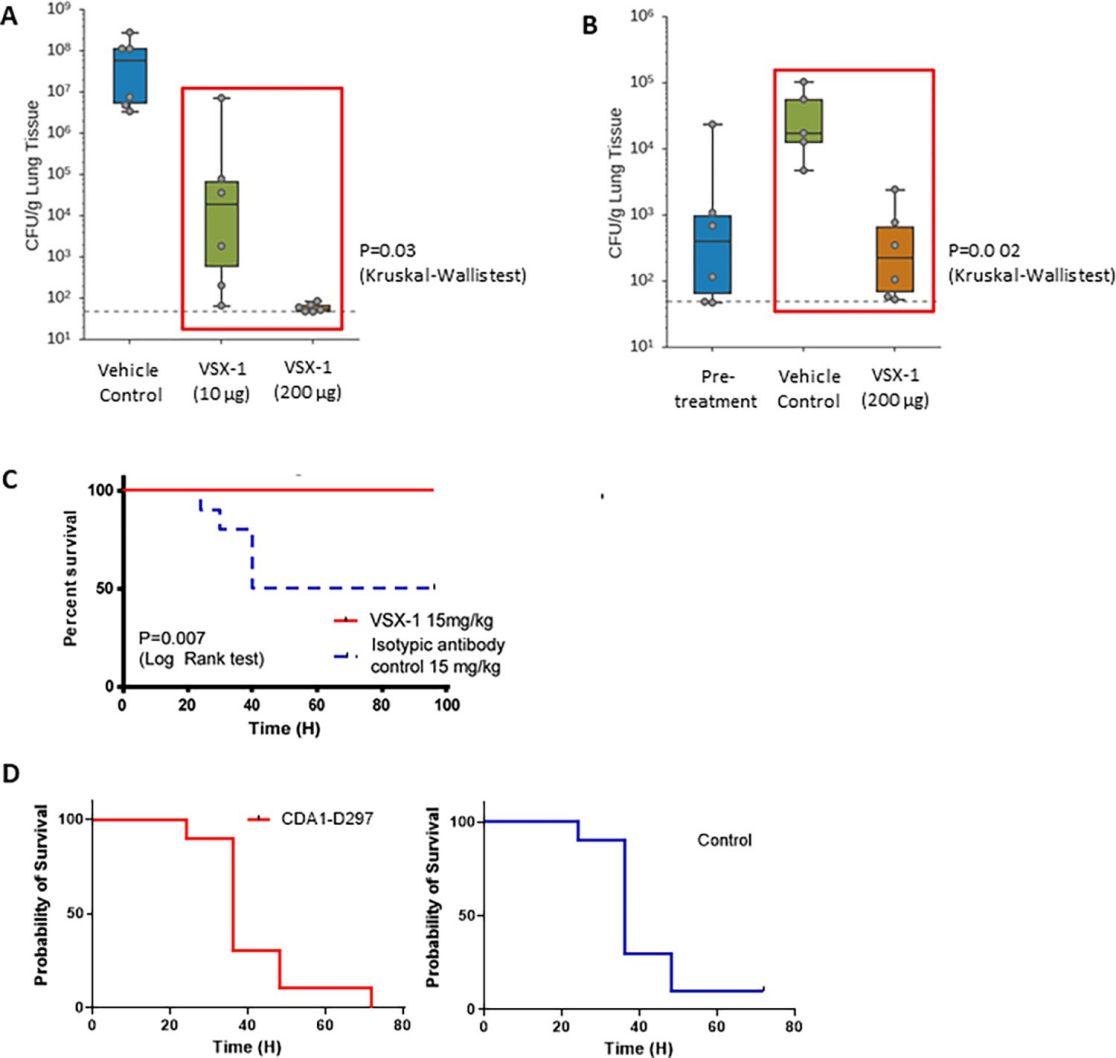
*Evaluation of conjugates in* in vivo *models of P*. *aeruginosa lung infection*. (**A**) Neutropenic animals were co-administered VSX-1 and bacteria (ATCC 27853). The CFU burden in the lung was measured at eight hours. Co-administration of 10 μg of VSX-1 resulted in a multi-log reduction in bacterial burden, with reduction to the limit of detection upon administering 200 μg of ADC. (**B**) Neutropenic animals were infected with *P*. *aeruginosa* (ATCC 27853) and were treated 1 hour post-infection with either vehicle or VSX-1 (200 μg). The CFU burden in the lungs was measured just before treatment (pre-treatment) and eight hours post-injection. (**C**) The acute lung infection model with *P*. *aeruginosa* PA14 (2x10^6^ CFU/animal, 1x10^6^ in each nostril), C57/Bl6, 10 animals per group (two experiments with 5 animal per group each time), intranasal inoculation, IP dosing (15 mg/kg) 4 hours post-infection.

Consistently with the *in vitro* data, VSX-1 demonstrated *in vivo* efficacy in multiple animal models of *P*. *aeruginosa* infection. A murine neutropenic lung infection model using *P*. *aeruginosa* (ATCC 27853) was previously described [38] and was initially employed to prove the concept that VSX-1 would work *in vivo* even in an immunocompromised host. Infected animals were treated intranasally with VSX-1 construct, resulting in a statistically significant reduction in the bacterial load in the lung when either administered at two different sites after a short interval (15 minutes) (**Fig 5A**) or when dosed therapeutically (**Fig 5B**). Building on this initial data, VSX-1 was evaluated in an immunocompetent murine model (**Fig 5C**) using the approach that we had previously reported and also used in this study to test the antibody VSX alone (**Fig 1B**): the acute lung infection model with the *P*. *aeruginosa* strain PA14 [6]. While VSX alone was able to significantly protect the infected mice, the survival of infected animals treated three hours post-infection with a single intraperitoneal dose of VSX-1 at 15 mg/kg (**Fig 5C**) was more pronounced (P = 0.04 and P = 0.007 respectively). In addition, our antibody alone showed no bacterial reduction in the absence of complement or PMN (S4 Fig). Taking all these results into account, an ADC with direct bactericidal activity seemed to be preferable to an antibody alone, particularly in an immunocompromised setting.

The immunocompetent model used for this experiment is aggressive, with ≥50% of control animals succumbing to infection within 48 hours of inoculation, as previously reported [6]. To confirm the contribution of the antibody component of VSX-1, a sham-conjugate was prepared using a non-*P*. *aeruginosa* targeting antibody (actoxumab, a human mAb against *Clostridioides difficile* [39]).

Overall, the superior protection conferred by the ADC (**Fig 5C**) compared with the mAb alone (**Fig 1B**) and the fact that the actoxumab-D297 conjugate showed no survival benefit in the immunocompetent lung infection model (**S6 Fig**) validated our selection of a *P*. *aeruginosa* surface-targeting antibody conjugated with an AMP. The protection observed with the VSX-1 conjugate was therefore highly encouraging and warranted the further exploration of this ADC as a potential treatment for *P*. *aeruginosa* lung infection.

### Optimization of the ADC: VSX-2

**Fig 6:**
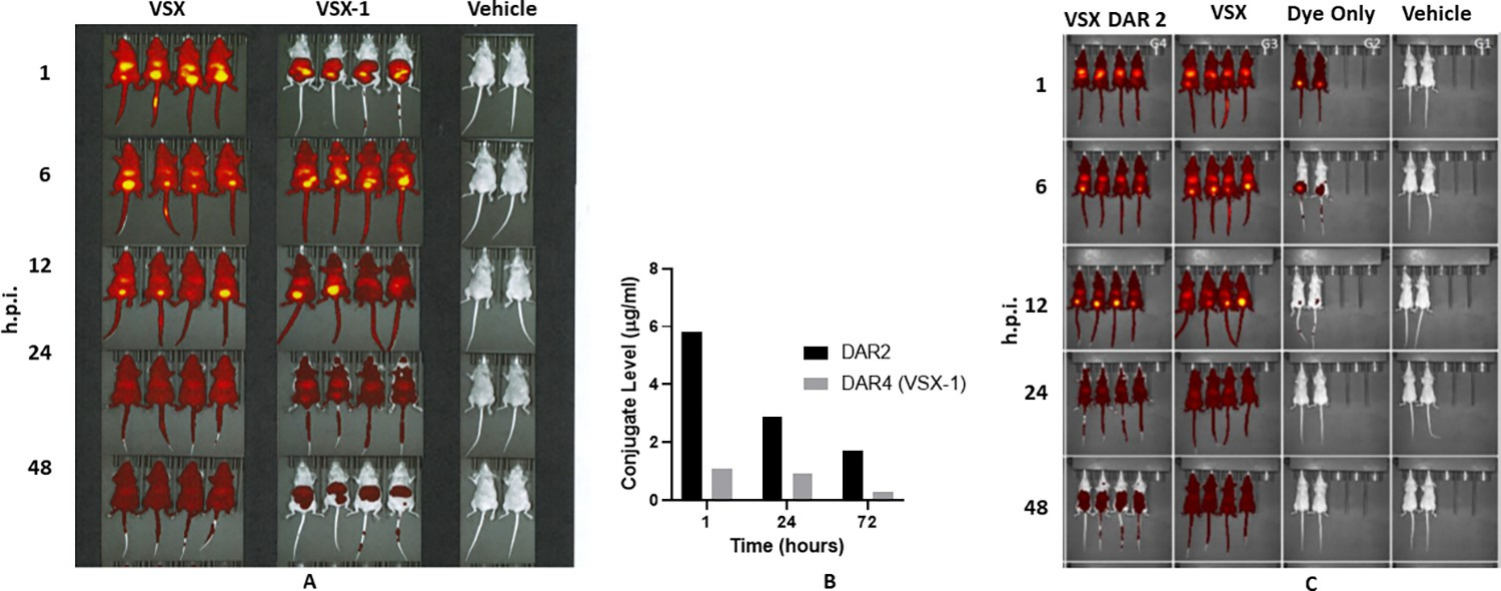
Biodistribution of VSX-1. (**A**) *In vivo* imaging of VSX-1’s biodistribution. Labeled VSX (the antibody alone) or VSX-1 (the ADC) was administered to animals IP and monitored by imaging up until 48 hours post-injection (h.p.i.). Both agents distributed throughout the animal (i.e. to all perfused organs) but VSX-1 had a shorter elimination time, with little remaining agent at 48 h.p.i. (**B**) Analysis of serum levels (using an ELISA) of VSX-1 (DAR = 4: grey bars) at 1, 24, and 72 hours post-injection, and DAR = 2 constructs (black bars). (**C**) The same imaging procedure as in (**A**); the ADC with a DAR = 2 demonstrates better biodistribution and a longer half-life.

The promising first generation VSX-1 construct was further evaluated *in vivo*. Despite demonstrating protection in *P*. *aeruginosa* infection models, we noted that VSX-1 displayed somewhat compromised biodistribution in mice, relative to the parent antibody, as well as a half-life of <24 hours (**Fig 6A**). Based on previous studies with ADCs, the AMP’s properties and DAR have been shown to compromise bioavailability *in vivo* with similar constructs [40]. Indeed, we found that a DAR of two between VSX and D297, while not as potent, demonstrated improved bioavailability in the mouse with increased circulating levels after 1, 24 and 72 hours post-administration, compared with VSX-1 **(Fig 6B).** This observation extended to other compartments as well, with increased bioavailability in the lung after 1h (**Fig 6C,** top left panel for VSX-DAR2 and **6A,** middle top panel for VSX-DAR4).

**Fig 7:**
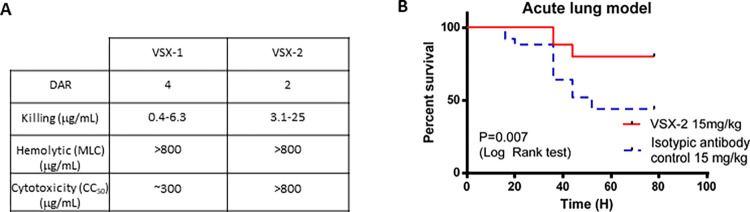
In vitro *and* in vivo *assessments of VSX-*2. (**A**) Activity of VSX-2 compared with VSX-1 with regard to bacterial killing, red blood cell hemolysis (mean lytic concentration (MLC)) and toxicity against mammalian cells (CC_50_). The bactericidal activity of VSX-2 is similar to (but slightly lower than) that of VSX-1, with a lower DAR, and with a similar inability to lyse RBCs or kill mammalian cells. (**B**) VSX-2 has similar activity *in vivo* in the acute lung infection model with *P*. *aeruginosa* PA14 (2 x 10^6^ CFUs/animal, 1 x 10^6^ CFUs in each nostril), C57/Bl6, 25 animals per group (five experiments with five animals per group each time), intranasal inoculation, and IP dosing (15 mg/kg) 4 hours post-infection.

Therefore, to optimize the biodistribution properties of VSX conjugates as a potential therapeutic, we carried out a focused set of studies on AMP charge, AMP hydrophobicity and DAR. Starting from the P297 sequence, we screened a set of charge variants and variants with reduced overall hydrophobicity. Unexpectedly, L-P369 (GGGKLLRKLKKSVKKRAKELLKKPRVIGVSIPL, containing 5 phenylalanine to leucine substitutions) emerged as a more potent peptide and, when conjugated to the VSX antibody, retained bactericidal activity and demonstrated little cytotoxicity against mammalian cells (**Fig 7A**). As with D297, P369 (containing either D- or L-amino acids) demonstrated potent MIC activity across several *P*. *aeruginosa* strains, including the MDR ATCC strain 2108 (MIC = 8 μg/ml). Indeed, VSX-2 (with a DAR of 2 with AMP D-369 ligated to the C-terminus of each HC) also demonstrated protection in the *P*. *aeruginosa* lung infection model (**Fig 7B**). Thus, it appeared that we could modify both the activity and the PK characteristics of the ADC through modification of the peptides’ characteristics and the DAR.

**Fig 8:**
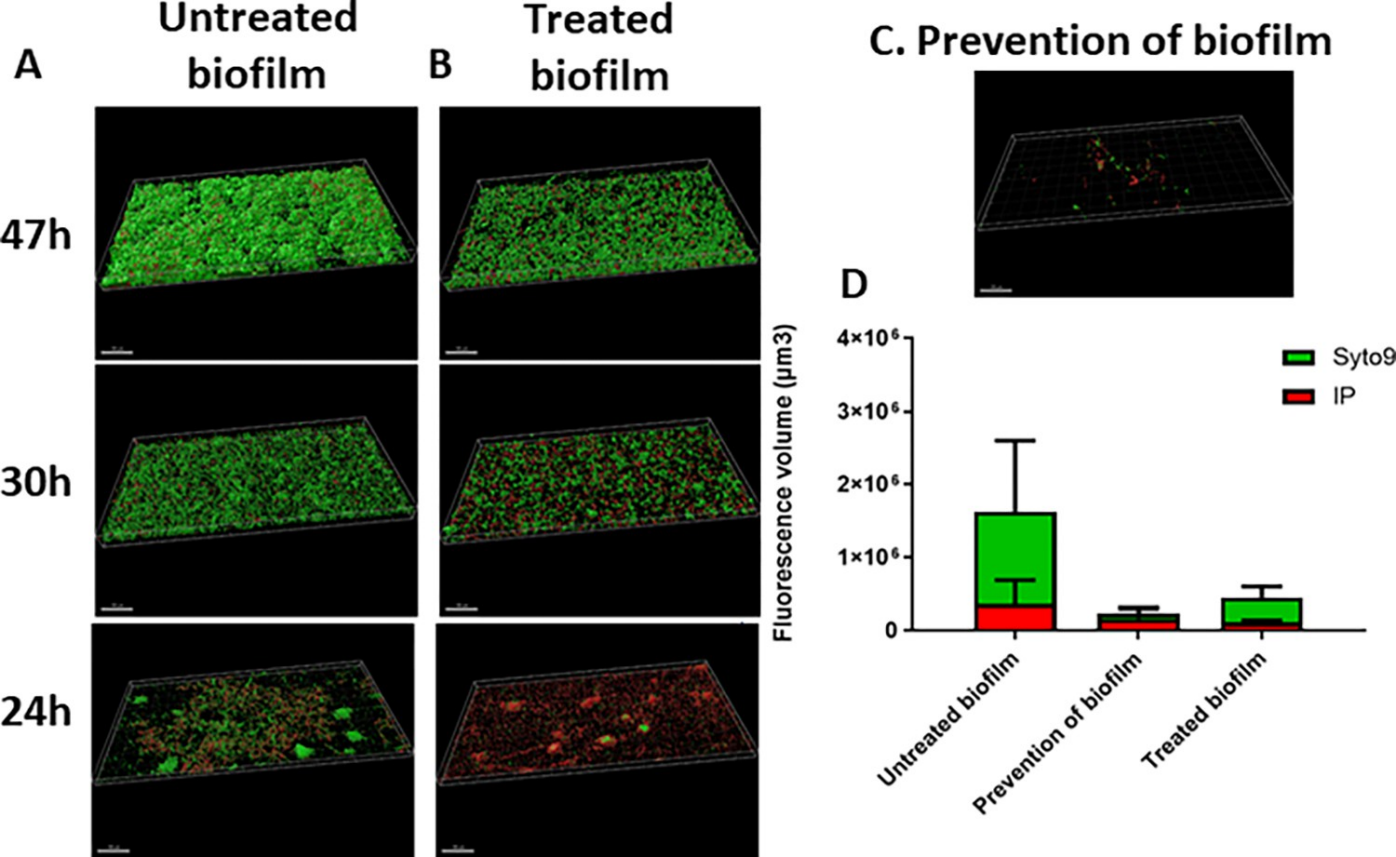
Prevention and treatment of *P*. *aeruginosa* PA14 biofilm formation in a dynamic model labelled with Syto9 (green) and PI (red) fluorochromes. Fig 8: Effect of preventive and curative treatments of *P*. *aeruginosa* PA14 biofilm in a dynamic model. (A-B). The live/dead bacteria within the biofilm were assessed with Syto9 (green = live) and propidium iodide (red = dead) fluorochromes. Fluorescence was measured at 47 h (top), 30 h (middle) and 24 h (bottom) as readouts to 3D-reconstruct the biofilm and determine its thickness in the control condition (A) and in the treatment condition with 6 μg/mL of VSX-2 (DAR2) for 1 h (B). (C) For the prevention of biofilm formation, fluorescence was measured after the injection of 6 μg/mL of DAR2 (VSX-2) during bacterial inoculation at time 0. (D) Fluorescence volume of the biofilm biomass, with the distribution of Syto9 and PI staining; scale bar = 100 μm. Fluorescent microscopy images of two adjacent fields from one sample, and calculation using Imaris software. The experiments were repeated three times.

Lastly, *P*. *aeruginosa* is also responsible for chronic infections, e.g. lung infections in cystic fibrosis patients and wound infections. In these chronic infections, the treatment is often complicated by the production of a biofilm by *P*. *aeruginosa*. Therefore, as a last step, we also tested the activity of our ADC VSX-2 against biofilm-grown *P*. *aeruginosa* in an inhibition/prevention setup and also against mature biofilms (an eradication/treatment setup, **Fig 8**). We used a dynamic model with a continuous, very low flow of minimal medium and *P*. *aeruginosa* biofilms grown for 48 hours at 37°C in flow chambers. While VSX-2 applied 24 hours after formation of a dynamic biofilm was able to totally eradicate *P*. *aeruginosa*, the efficacy of the treatment decreased overtime (**Fig 8A and 8B**). Prevention of biofilm formation by our ADC (injection of VSX-2 in the flow cell system during bacterial inoculation) was highly successful in our model (**Fig 8C and 8D**)

Taken as a whole, the results of this study describe a new approach for treating bacterial infections, including those caused by multidrug-resistant strains through the use of an ADC acting at the outer membrane of Gram-negative organisms, specifically *P*. *aeruginosa*. Our selected antibody targets a conserved glycan structure on the surface of *Pseudomonas aeruginosa* [16]. It has been engineered in an ADC format with direct bactericidal activity. Unlike ADCs that have been constructed for oncology indications [41] and more recently for Gram-positive organisms [42], internalization and release of the AMP is not required for activity: we present here the construction of an ADC that acts exclusively at the outer membrane. The resulting ADC demonstrates significant *in vitro* activity against a variety of strains of *P*. *aeruginosa* and has demonstrated *in vivo* efficacy in an aggressive lung infection model.

While these data demonstrate initial proof-of-concept that an ADC approach can work for a Gram-negative organism, there are some limitations to this study. First, while our preliminary work indicated that the frequency of bacterial resistance to the peptides and ADCs is low (~10^−8–10^, roughly equivalent to colistin), additional work will be required to further characterize the resistant mutants. Secondly, while the biodistribution of the VSX-1 ADC is sufficient to exhibit *in vivo* activity and is significantly improved with the redesigned VSX-2, neither construct exhibited biodistribution or PK properties comparable to the antibody alone. Lastly, additional studies and other ADC constructs are required to determine whether Gram-negative bacteria other than *Pseudomonas* can be targeted with our approach.

Overall, our ADC strategy augmented the bactericidal activity of specific antibodies. By bringing together two optimized components (a specific human anti-*P*. *aeruginosa* antibody and an optimized AMP), the resulting ADC had therapeutic properties superior to those of either component alone. The data and approach presented here offer an alternative strategy for the development of antimicrobials and complement ongoing efforts with small molecules and biologics [43].

## Materials and methods

### P. Aeruginosa strains

All the strains used in the study and their origin are listed in S1 Table.

*VSX Antibody*. The fully humanized antibody VSX targets the inner core of *P*. *aeruginosa* LPS and specifically the phosphodiheptose. A complete description of this antibody is presented by Elli, *et*. *al*. [16].

### Sortase tagging

A sortase A recognition sequence (LPETGGSG) was placed at the C-termini of either the heavy chain (DAR_theoretical_ = 2) or both the heavy and light chains (DAR_theoretical_ = 4) of the VSX mAb. Prior to ligation, the VSX antibody was buffer-exchanged from 1x PBS into sortase buffer (150 mM NaCl/50 mM Tris, pH 7.5), using 30 kDa spin diafiltration units (Amicon Ultra 15). Sortase A was from BPS Bioscience (San Diego, CA). VSX was ligated with the transpeptidase enzyme and a GGG-sortase donor peptide (added to the reaction as an aqueous solution at 10 or 20 mg/ml), thereby replacing the GGSG sequence on the antibody with full-length peptide. Ligations were performed in sortase buffer, using 20 mol of peptide per mol VSX antibody (which was at a concentration of 1.5 mg/ml), 10 mM CaCl_2_, 5.8 μg/ml sortase A. Samples were kept in the dark at room temperature for 18 hours, followed by quenching via dilution to 10 ml total volume in PBS and immediate purification by Protein A FPLC. Conjugation efficiency was determined by Q-TOF mass spectrometry, using a reduced antibody prepared by heating 5 μg of sample at 65°C for 15 min in 10 mM DTT.

### SEC-HPLC

VSX and VSX conjugate were dissolved at 1 mg/ml and 10 μl were injected onto a BioSep SEC-s3000 Phenomenex column (300 x 7.8 mm) running at 1 ml/min in 0.1M NaH_2_PO_4_ buffer at pH 3 on an Agilent 1100 series system with 214 nm UV monitoring.

### Assessment of AMP Stability in serum

Normal human serum (NHS, Sigma S-7023) was thawed, diluted in water, and centrifuged at 13,600 x g for 10 minutes. The supernatant was warmed to 37°C in a water bath. Twenty microliters of each test article were placed in a 2.0 ml round bottom microfuge tube. Two milliliters of diluted NHS were added to each tube, which was immediately vortexed, followed by transfer of 200 μl to a fresh microfuge tube placed at 37°C in a rotating rack. Samples were harvested and processed at various time-points up to 6 hours by quenching with 40 μl of 15% trichloroacetic acid (TCA), chilling on ice for 15 minutes, centrifuging at 13,600 x g for 10 minutes, and storing the supernatant at -20°C until analysis.

### Assessment of Antimicrobial activity

MICs and a killing assay were performed in this study. MICs were determined according to CLSI guidelines, using 2-fold serial compound dilutions in 96-well microtiter plates. Briefly, compounds were diluted in water across a mother plate and then 2 μl were stamped to assay plates—one plate for each strain to be tested. Bacterial strains were sub-cultured overnight on agar plates at 37°C. Overnight plates were used to prepare 0.5 McFarland cultures in 0.85% saline. These concentrated cultures were diluted 1:200 in growth media to approximately 5 x 10^5^ cells/ml. All assay plates received 100 μL diluted culture per well. All plates were placed at 37°C overnight. After 18 hours, the plates were assessed using a mirrored plate reader and incandescent light. The MIC is defined as the lowest concentration of compound that inhibits growth by at least 80%. Wells at and above the MIC should appear devoid of growth when visualized.

To assess microbial killing in the killing assay, bacterial cells were grown aerobically overnight on agar plates at 37°C. Overnight plates were used to seed 30 ml cultures of growth media in 250 ml vented flasks. Cultures were grown aerobically at 37°C with shaking at 150 rpm. Growth was monitored at A_600_, and bacterial cells were harvested at mid-log growth. Ten milliliters of culture were pelleted at 4000 x g for 10 minutes and washed once with PBS + 1% BSA (PBSA) prior to resuspension in 2 ml of PBSA. The concentrated culture was used to seed 6 ml tubes of PBSA to an OD giving a concentration of 1 x 10^8^ cells/ml. Cultures were diluted to 1 x10^4^ cells/ml in PBSA. Test substances were diluted in PBSA, and 50 μL per concentration tested were loaded into a 96-well polypropylene microtiter plate. Fifty microliters of diluted culture were added to all test wells and all no-compound control wells. Plates were shaken and then incubated at 37°C for 90 minutes without shaking. Ten microliters from each assay well were plated onto agar plates and incubated at 37°C overnight. Percent killing was determined by the CFU for test wells vs. the CFU for no-compound control wells. The EC_50_ reported is the lowest concentration of a compound which causes > 50% colony reduction compared with the no-compound control for the strain being tested.

### RBC Hemolysis assay

Test substances were diluted 2-fold in water across a 96-well polypropylene mother plate, leaving one no-compound control. Two microliters from each well were stamped onto a 96-well polystyrene assay plate. A red blood cell suspension was made by mixing 0.2% defibrinated sheep’s blood (Hardy Diagnostics DSB100) in PBS. All assay plates received 100 μl/well of the red blood cell suspension. Plates were then incubated at 37°C overnight. The titer for RBC hemolysis was defined as the lowest concentration of compound that completely prevented the formation of a red blood cell pellet perceptible by eye.

### Mammalian cell cytotoxicity

On day one, cells from an established 293T human cell line were seeded onto 96-well flat-bottom white plates (10,000 293T cells/well). On day two, dilution plates were prepared by diluting compound in cell growth medium to two times the final starting concentration and then carrying out serial two-fold dilutions across the plate. Medium from the cell growth assay plates was then aspirated, and 50 μl from the compound dilution plates were transferred to the assay plates. Fifty microliters of fresh media were then added to all the wells. The plates were incubated at 37°C, 5% CO_2_ for three days. Then, CellTiter Glo (Promega G7570) was reconstituted and mixed 50:50 with growth media. Media from the assay plates was aspirated, and 100 μl of CellTiter Glo/growth media mix was added to all wells. After five minutes, luminescence was read, and% inhibition *vs*. compound concentration was plotted. The CC_50_ was defined as the cytotoxic concentration reducing the viable cell number by 50%, compared with cells in media lacking the test substance.

### LPS Neutralization assay

A cell-based LPS neutralization assay was developed and optimized. The HEK-Blue LPS detection Kit2 (Invivogen) was used to investigate the ability of VSX to neutralize the endotoxin activity of extracted *P*. *aeruginosa* LPS in HEK-Blue cells. Endotoxins present in the media or standard are sensed by TLR4, leading to the activation of NF-κB and the concomitant production of SEAP in the supernatant. When supernatant is combined with QUANTI-Blue (which contains a SEAP chromogenic substrate), a purple/blue color appears and can be quantified by measuring the absorbance at 620–655 nm and extrapolating against a standard curve. Endotoxin units (EUs) of 0.5 EU/ml were used to define the LPS concentration added alone or pre-mixed with VSX (0.185–10 μM), and activation of the NF-κB was assessed. The CDA1 mAb does not target bacteria and was used as a control in the neutralization assays.

### The mixed microbial assay

Bacterial strains were grown overnight on agar plates at 37°C. Overnight plates were used to establish 0.5 McFarland Cultures in 6 ml PBS (approximately 1 x 10^8^ cells/ml). Concentrated cultures were diluted to 1 x 10^4^ cells/ml in PBS. Ten microliters of each of the diluted cultures were plated onto BAPs to determine the initial concentration, check for purity, and establish the strain morphology. One milliliter of each diluted culture was combined (giving a total of 3 ml) and the volume was brought to 10 ml with PBS (1 x 10^3^ cells/ml of each strain). Twenty-five microliters of the mixed culture were spread on a BAP, to establish the CFU/ml for each strain at t = 0. Test substances were serially diluted 4-fold in PBS with a final volume of 200 μl in 2 ml round-bottom Eppendorf tubes. A no-compound control was included. Two hundred microliters of mixed bacterial culture were added to all assay tubes. Tubes were vortexed, and 50 μl from each tube were immediately spread on separate BAPs. Assay tubes were incubated at 37°C, rotating between timepoints. The plating procedure was repeated at 1-hour intervals for two hours, and all plates were incubated at 37°C overnight. The following day all plates were counted by noting the CFU for each strain (distinguished morphologically), and results were plotted as the percentage killed vs. a no-compound control.

### Resistance assessment, by MIC

Resistance to D297 was assessed using two, complementary methods. The first (standard) protocol involved plating a high-density culture on selective plates at various multiples of the MIC for D297 and, as a reference, colistin. Overnight cultures were brought to an OD of 3.0 at a wavelength of 600 nm (10^9^–10^10^ CFU/ml), and 100 μl of concentrated culture were plated in quadruplicate onto selective plates containing compound at 2, 4, and 8 x MIC. Additionally, 100 μl of 10^−7^, 10^−8^, and 10^−9^ serial dilutions of the concentrated cultures were plated in quadruplicate on non-selective plates to calculate the CFU/ml. All plates were incubated at 37°C and counted at 24 and 48 hours. Resistance rates were calculated. To confirm resistance, colonies growing at 2 x MIC and higher were re-plated onto selective plates.

Secondly, resistance to peptide D297 was determined using Pranting et al.’s microdotting procedure [44]. Solid-phase MICs for test substances on tryptic soy agarose plates were determined by plating 100 μl of a bacterial culture with > 1 x 10^9^ CFU/ml onto selective plates containing the test compound at concentrations equal to various multiples of a broth MIC previously determined using CLSI standards. The concentration of the plate leading to an 80% reduction of CFU/ml vs. a non-compound control plate defined the solid-phase MIC. Selective tryptic soy agarose plates were prepared at concentrations of 0, 2, 4, and 8 times the solid-phase MIC for D297 and, as a reference, colistin. Bacterial test strains were grown overnight, rotating at 37° C in 40 separate 10 ml Pyrex screw-capped tubes containing 3 ml Meuller Hinton Broth II, cation adjusted (MHB). Overnight cultures were spun down and re-suspended in Tryptic Soy Broth (TSB) to a density of approximately 4.8 x 10^9^ CFU/ml as determined by densitometer. Serial dilutions of representative cultures were plated to confirm cell concentrations. All cultures were plated on selective and non-selective plates by dropping 5 μl of culture onto agarose plates and letting the drops absorb. Plates were incubated at 37° C overnight. The mutation rate was calculated using the P_o_ equation -[ln(P_o_/P_tot_]/N, where P_o_ is the number of cultures (spots)with no mutants, P_tot_ is the number of cultures, and N is the number of bacteria applied in each spot.

### Generation of resistant mutants

Plates generated during the resistance assessment using both the standard and Pranting protocols (see above) were used to isolate mutant strains of *P*. *aeruginosa* 27853 with elevated MICs to peptide D297. One colony from the standard protocol and 5 colonies from the Pranting protocol grew at 4 x the agarose MIC for wild-type *P*. *aeruginosa* 27853. These six colonies were picked and mixed with PBS. Five-microliter drops containing approximately 2-4x10^8^ cells were re-plated onto D297 selective plates (1–4 x MIC), colistin-selective plates (0.5–4 x MIC), and non-selective plates to determine whether the mutations conferred true resistance and whether any cross-resistance to colistin had been created. Plates were incubated at 37°C overnight. Of the six potential mutants, 4 showed confirmed growth at 2 x the MIC and 1 showed confirmed growth at 4 x MIC on D297-selective plates. Only one of the 6 isolates grew at 2 x MIC on colistin-selective plates. Wild-type control strains did not grow at 2 x MIC for either D297 or colistin.

### Competitive fitness assay

A competitive fitness assay was run with the wild-type *P*. *aeruginosa* 27853 and the resistant mutant strain. Both isolates were grown overnight on agar plates, followed by independent culture for 24 hours in 35 ml of MHB in flasks. Overnight cultures were used to constitute 0.5 McFarland cultures, using a densitometer. 50 μl of each 0.5 McFarland culture was plated onto BAPs. Cultures were then mixed so that 3, 30 ml cultures in flasks would contain approximately 1 x 10^6^ of each cell type (1:100 dilution), 1 x 10^5^ of each cell type (1:1,000 dilution), and 1 x 10^4^ of each cell type (1:10,000 dilution). At 24 hours, we plated 50 μl of serial dilutions of McFarland cultures and mixed cultures onto BAPs to determine the CFU/ml. All plates were at 37°C overnight. On the following day, we counted all plates for CFU/ml and then re-plated the mixed bacterial flasks by diluting each mixed culture to 0.5 McFarland, diluting to 1 x 10^5^ cells/ml, and plating 50 μl of the diluted cultures onto BAPs. All plates were incubated at 37°C overnight and then read for CFU/ml.

To determine whether there was a phenotypic difference between wild-type *P*. *aeruginosa* strain ATCC 27853 and its resistant mutant, both strains were plated on eight different types of selective and non-selective agar mediums. On one type of plate (TSA with 5% sheep’s blood) there was a discernible difference in the colony morphology between the wild-type and mutant strains, with the later exhibiting a rougher colony morphology; this enabled the use of these BAPs for the determination of the relative fitness of the two strains.

To assess relative fitness, we used the method described by Lenski et al. [45] to estimate the selection coefficient on a genotype *s* from competition data (where relative fitness is given by 1+*s*). The growth parameter for a strain is the number of doublings that it experiences over a given period of time. As such, the selection coefficient on the focal strain is defined as follows:

$$s_{i}=\frac{No.\;of\;doublings\;of\;focal\;strain}{No.\;of\;doublings\;of\;wild\;type\;strain}-1$$

Note that *s*_*i*_ is a unitless parameter.

### Serum stability assay

Normal human serum (NHS) (Sigma S-7023) was thawed, diluted in water and centrifuged at 13,600 x g for 10 minutes, and the supernatant was warmed to 37°C in a water bath. Twenty microliters of each test article at 10 mg/ml were placed in a 2.0 mL round-bottomed microfuge tube. Two milliliters of NHS (25% in dH_2_O) were added to each tube. The tubes were immediately vortexed, and 200 μl were transferred to a fresh microfuge tube with 40 μl of 1% trichloroacetic acid (TCA). Assay tubes were placed at 37°C in a rotating rack. Additional samples were similarly harvested and processed at t = 30, 60, 120, 240, and 360 minutes. TCA tubes were placed on ice for 15 minutes and then centrifuged at 13,600 x g for 10 minutes. Supernatant from each tube was collected and frozen at -20°C for analysis. Mass spectrometry was used to quantify the percentage of test substance still intact.

### Opsonophagocytic killing assay

Opsonophagocytic killing assays were performed in 2.0 ml Eppendorf tubes and a total volume of 400 μl. Four components were added in rapid succession in equal (100 μl) volumes: test substances, bacterial culture, PMNs, and human complement. Test substances were diluted in GVB +Ca +Mg (Boston Bioproducts #IBB-300X) and kept on ice until used. Overnight bacterial cultures were grown in Columbia Broth + 2% NaCl (CSB) at 37°C, with rotation at 250 rpm. Cultures were diluted to an OD at 650 nm of 0.4 in GVB and then diluted again (1:200) in GVB to give a final assay concentration of 1.5 x 10^7^ CFU/ml. Human PMNs were isolated with EasySep (StemCell Cat. # 19666) as per the protocol for peripheral blood. PMNs were resuspended in GVB to 1 x 10^7^/ml (1 x 10^6^ PMN/tube). For no-PMN control tubes, GVB was used. The complement used was 20% MN8-absorbed Human C’ (single source) in GVB. The complement underwent further absorption using 200 μl C’ with 800 μl of a bacterial suspension in GVB at an OD at 650 nm of 1.0. Absorption was performed at 4°C for 30 minutes. Cells were spun out, and the process was repeated; the supernatant was used to resuspend a cell pellet from 800 μl cell culture. The cells were spun out, and the final supernatant was filtered using 0.22 μm spin filters. The final complement source was 2x absorbed, 20% human complement. A small aliquot was heat-inactivated at 56°C for 30 minutes, giving a no-complement control. The components as described were combined, and 25 μl from each assay tube were removed for a t = 0 CFU determination. The assay tubes were capped and placed at 37°C for 90 minutes with end-over-end rotation. The removed samples were serially diluted 1:10 in TSB/Tween, and 10 μl of the 1:10 and 1:100 dilutions were plated on TSB BAPs by allowing the sample to run down the vertical plate almost to the edge. After a 90-minute incubation, the plating procedure was repeated. All plates were incubated overnight at 37°C, and CFU/ml calculations were performed for t = 0 and t = 90 minutes.

### The neutropenic mouse model

Animal experiments were performed in accordance with our Institutional Animal Care and Use Committee’s rules. CD1 mice were supplied by Charles River (Margate, UK) and were specific-pathogen-free. Male mice weighed 11–15 g on receipt and were allowed to acclimatize for at least 7 days. The mice were housed in sterilized, ventilated, individual cages that exposed the animals at all times to HEPA-filtered sterile air. Mice were rendered neutropenic by immunosuppression with cyclophosphamide at 200 mg/kg four days before infection and 150 mg/kg one day before infection, by intraperitoneal (IP) injection. With this immunosuppression regime, neutropenia started 24 hours after administration of the first injection and continued throughout the study. *P*. *aeruginosa* strain ATCC 27853 was used to assess *in vivo* protection. For infections, animals were first anesthetized with an IP ketamine/xylazine anesthetic cocktail (90 mg/kg ketamine & 9 mg/kg xylazine) at ~15 ml/kg. Anesthetized mice were infected with a 0.04 ml inoculum by intranasal instillation (20 μL per nostril and a 5-minute interval between each nostril administration) and were kept in an upright position on a string rack for ~10 minutes post-infection. The inoculum concentration was 2.83 x 10^5^ CFU/ml (~1.1 x 10^4^ CFU total inoculum). VSX-1 or controls were administered either intranasally (IN) or IP. The clinical condition of the animals was monitored, and animals that succumbed to the disease were euthanized. The study was terminated ~24.5 hours post-infection, when most of the vehicle mice were displaying significant clinical symptoms, after which the clinical condition of all remaining animals was assessed. After being euthanized by pentobarbitone overdose, the mice’s weights were determined before the lungs were removed and weighed. Lung samples were homogenized in ice-cold, sterile 1x PBS using a Precellys bead beater; the homogenates were quantitatively cultured onto *P*. *aeruginosa* selective agar and incubated at 37°C for 16–24 hours before the colonies were counted. Data were analyzed using StatsDirect software (version 2.7.8). The non-parametric Kruskal-Wallis test was used to test all pairwise comparisons (Conover-Inman) of the tissue burden data.

### The acute lung infection model

The acute lung infection model was performed as described previously [6], with some minor modifications. Briefly, after general anesthesia (IP injection of ketamine and xylazine) of C57/BL6 female 6–8 week old mice, 10 μL (1x10^6^ CFU) of the reference strain *P*. *aeruginosa* PA14 were inoculated into each nostril to induce an acute lung infection. The inoculum was prepared from a dilution of an overnight culture of PA14 grown in Luria Bertani broth. Mice were monitored for four days and were euthanized when they showed imminent signs of mortality, including ruffled fur, lethargy, shaking, high respiratory rate, inability to move when touched, or inability to right itself after being placed on its side. For the protection assays, the ADC or the control compound were injected IP at 15 mg/kg three hours after the bacterial challenge. For each experiment, there were five animals per group. Experiments were repeated twice with VSX and VSX-1 and five times with VSX-2.

### In Vivo imaging

CR female NCI Ath/nu mice were placed on a special "RD D10012Mi" diet for 7 days prior to the start of the study and for the duration of the study. The animals were randomized into treatment groups based on the Day 1 bodyweight, with an age at start date of 8 to 12 weeks. VSX was conjugated with IR800 dye (LiCor, Lincoln, NE) and then used in sortase reactions to make VSX-1. A vehicle control (0.9% saline) and a dye-only control were also employed. A 5 mg/kg dose was used, and substances were injected IV. Whole-body imaging (dorsal and ventral) was performed at 1, 6, 12, 24, 48 and 100 hours post-IV dosing.

### Flow Cells/Biofilm experiments

*P*. *aeruginosa* biofilm were grown in a dynamic model with continuous, very low flow of minimal medium (MM: 62 mM potassium phosphate buffer, pH 7.0, 7 mM (NH_4_)_2_SO_4_, 2 mM MgSO_4_, 10 mM FeSO_4_) containing 0.4% glucose and 0.1% casamino acids.

In this dynamic model, *P*. *aeruginosa* biofilms were grown for 48 hours at 37°C in flow chambers (IBI Scientific, UK). The system was sterilized by pumping a 0.2% hypochlorite solution through for 1 hour, using a peristaltic pump. After a rinse with sterile water, MM was introduced for 1 hour at a rate of 20 mL/h for system stabilization. Bacteria at a concentration of 5x10^8^ CFU/mL were injected into flow cell chambers, which were flipped upside down without flow for 2 hours. The pump was turned on to provide a constant rate of 0.2 mL/h of MM for 48 hours. For the preventive approach, bacteria were inoculated with VSX-2 (6 μg/mL). For the treatment experiment, we injected VSX-2 (6 μg/mL) for 1 hour at three different times (24, 30 and 47 h). After 48 h, a Live/Dead BacLight bacterial viability kit (Molecular Probes) at a Syto-9:propidium iodide ratio of 1:5 was injected. Next, stained biofilms were observed using a LMS 710 NLO confocal laser scanning microscope (Zeiss). The homogeneity of the samples was checked by traversing the observation field, and the most representative area was chosen for the acquisition of the image. 3D reconstructions and fluorescents volumes were generated using Imaris software. The experiment was repeated three times.

## Supporting information

S1 Fig CD analysis of P297. Circular dichroism spectra of P297 in an increasing hydrophobic buffer. The graph shows an increase in alpha-helical character with hydrophobicity.
(TIF)

S2 Fig (D)-P297 is more stable than (L)-P297 in the presence of human serum. (left) Representative total ion chromatograms of P297 at t = 0 (top) and after 60 minutes in 10% normal human serum (NHS). Intact peptide remaining was quantified by integration of the extracted ion current for (L)- or (D)-P297. (right) Sixty-minute time course of degradation of P297 variants in the presence of 10% normal human serum (NHS). Values were normalized against the starting peptide content (time = 0 min).
(TIF)

S3 Fig Analysis of the conversion of VSX to DAR2 and DAR4. (A) Q-ToF analysis of the heavy chain of the starting material and the sortase-ligated D-P297 product for sortase acceptors on both the heavy and light chains (top two panels), and for sortase acceptors on the heavy chains only (bottom two panels). Assuming the unbiased detection of starting material and product, 88.7% of the heavy chain for DAR4 material was ligated to D-P297. (B) Coomassie-stained gel analysis, showing the conversion of the starting material to the desired D-P297 ADC product. Lanes 1 and 2 are the starting material and the product for D-P297 ligation to both heavy and light chains. A second gel containing lanes 3 and 4 shows respectively the starting material and the product for D-P297 ligation to the heavy chains only (the light chain in the antibody starting material was wild-type and so lacked the sortase acceptor recognition sequence). (c) Calculation of the average DAR for constructs, using deconvoluted spectra: with a theoretical DAR of 4, the experimentally determined mean DAR was 3.8; for the theoretical DAR of 2, the experimentally determined mean DAR was 1.8.
(TIF)

S4 Fig The mixed microbial killing assay. *P*. *aeruginosa* ATCC 2108 (blue), *E*. *coli* ATCC 25922 (red) and *K*. *pneumoniae* ATCC 33495 (green) were co-cultured overnight, diluted, and then exposed to the specified agent for two hours. Killing was assessed visually. (left) Peptide D297 alone killed *P*. *aeruginosa* and *E*. *coli*, confirming its lack of specificity. (middle) In the absence of complement or immune cells, VSX alone had no killing effect. (right) VSX-1 demonstrated the rapid, specific, complete killing of *P*. *aeruginosa*. Little killing of *E*. *coli* was found, which confirmed the specific activity of the ADC.
(TIF)

S5 Fig Neutralization of *P*. *aeruginosa* LPS activity *in vitro*. Binding of either VSX (blue) or actoxumab (grey) to LPS in an optimized, cell-based LPS neutralization assay. The HEK-Blue LPS detection Kit (Invivogen) was used to investigate the ability of VSX to neutralize the endotoxin activity of extracted *P*. *aeruginosa* LPS on HEK-blue cells. In this case, endotoxin present in the medium or the standard is sensed by TLR4, leading to the activation of NF-kB and the production of SEAP in the supernatant. When the supernatant is combined with QUANTI-Blue, this activation can be visualized and compared with a standard curve. 0.5 EU/ml *P*. *aeruginosa* LPS serotype was used for all assays.
(TIF)

S6 Fig Control experiment to Fig 5C: in our acute lung infection model, mice were injected with either (i) D297 attached to actoxumab (CDA1) to measure the protective effect of the AMP alone (red curve, on the left) or (ii) with a saline solution (blue curve, on the right). No difference was found.
(TIF)

S1 Table Strains used in the study. (TIF)
