# Supplementary figures and images for: Development of an antibody fused with an antimicrobial peptide targeting Pseudomonas aeruginosa: A new approach to prevent and treat bacterial infections
Source: PLoS Pathog. 2023 Sep 7;19(9):e1011612. doi: 10.1371/journal.ppat.1011612 (PMC10508631; doi:10.1371/journal.ppat.1011612)

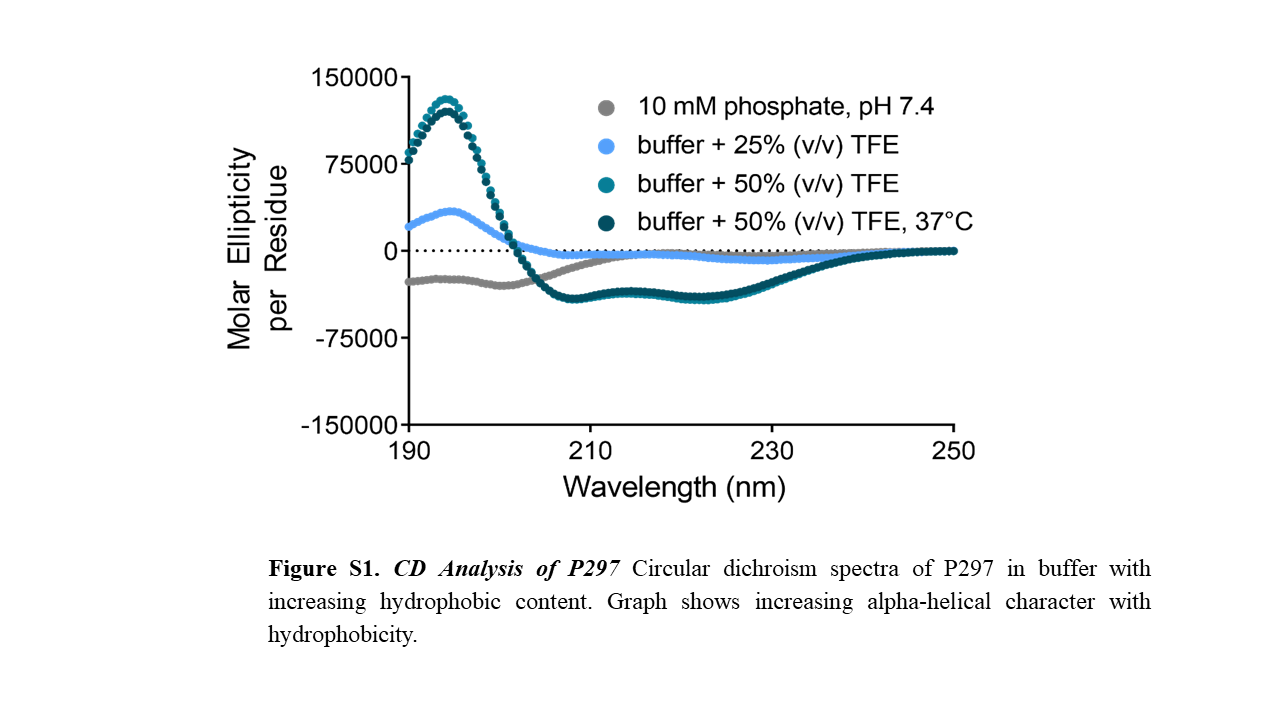

Supplement: S1 Fig — Circular dichroism spectra of P297 in an increasing hydrophobic buffer. The graph shows an increase in alpha-helical character with hydrophobicity. (TIF) [file ppat.1011612.s001.TIF]

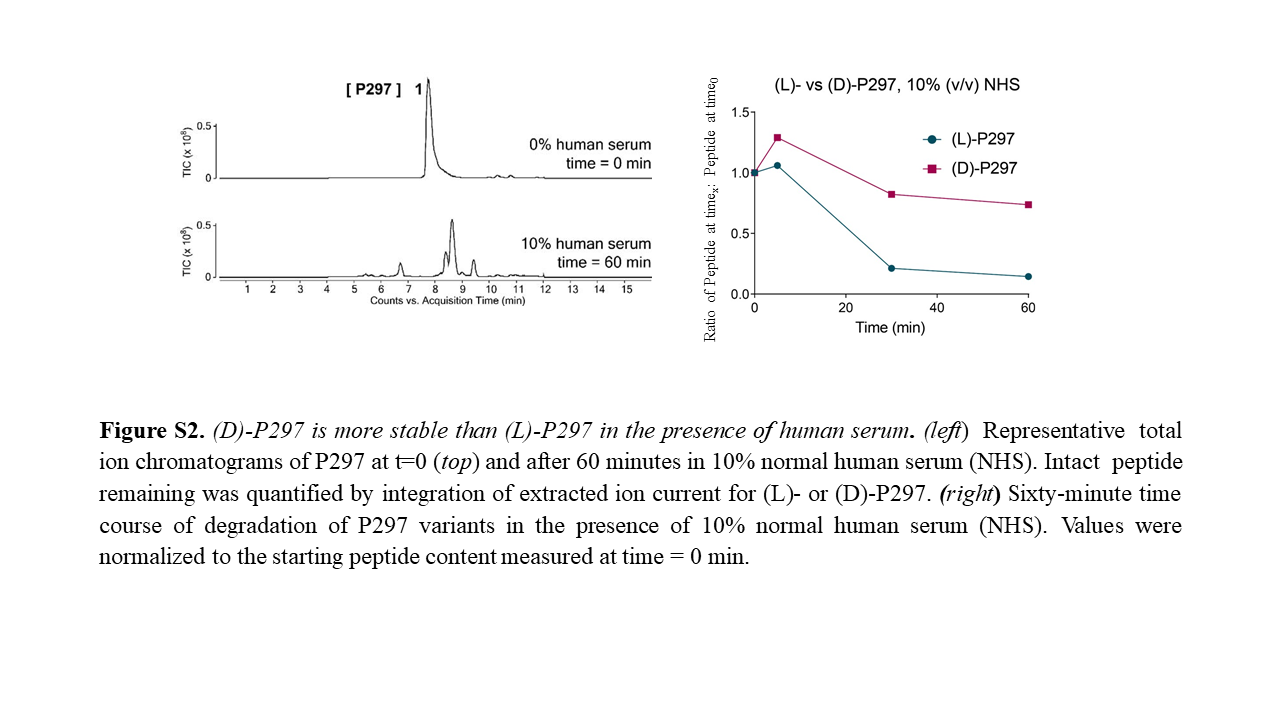

Supplement: S2 Fig — (left) Representative total ion chromatograms of P297 at t = 0 (top) and after 60 minutes in 10% normal human serum (NHS). Intact peptide remaining was quantified by integration of the extracted ion current for (L)- or (D)-P297. (right) Sixty-minute time course of degradation of P297 variants in the presence of 10% normal human serum (NHS). Values were normalized against the starting peptide content (time = 0 min). (TIF) [file ppat.1011612.s002.TIF]

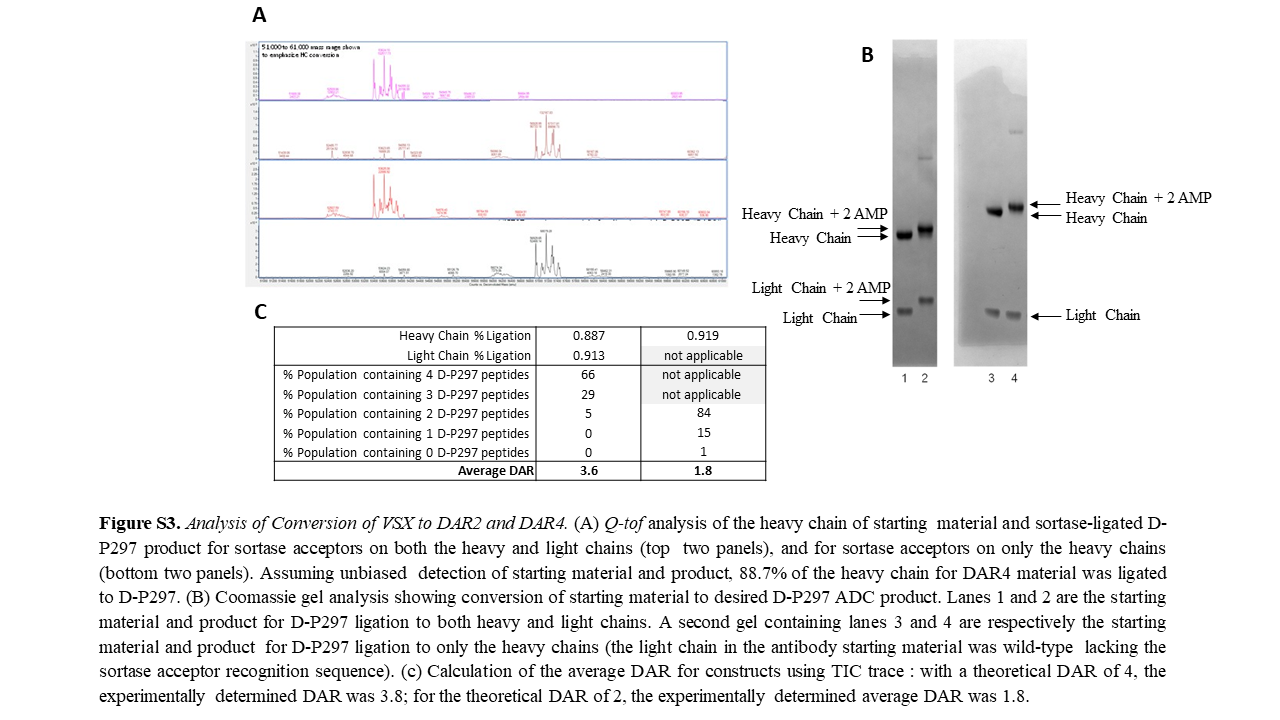

Supplement: S3 Fig — (A) Q-ToF analysis of the heavy chain of the starting material and the sortase-ligated D-P297 product for sortase acceptors on both the heavy and light chains (top two panels), and for sortase acceptors on the heavy chains only (bottom two panels). Assuming the unbiased detection of starting material and product, 88.7% of the heavy chain for DAR4 material was ligated to D-P297. (B) Coomassie-stained gel analysis, showing the conversion of the starting material to the desired D-P297 ADC product. Lanes 1 and 2 are the starting material and the product for D-P297 ligation to both heavy and light chains. A second gel containing lanes 3 and 4 shows respectively the starting material and the product for D-P297 ligation to the heavy chains only (the light chain in the antibody starting material was wild-type and so lacked the sortase acceptor recognition sequence). (c) Calculation of the average DAR for constructs, using deconvoluted spectra: with a theoretical DAR of 4, the experimentally determined mean DAR was 3.8; for the theoretical DAR of 2, the experimentally determined mean DAR was 1.8. (TIF) [file ppat.1011612.s003.TIF]

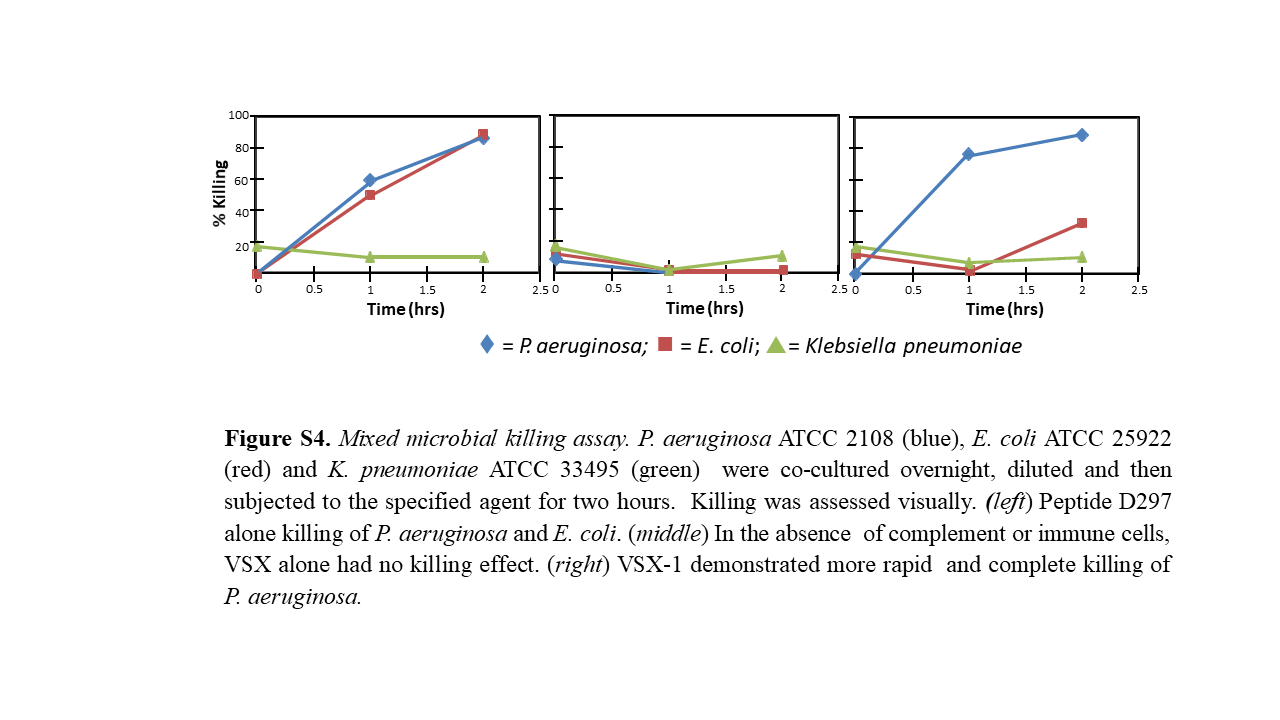

Supplement: S4 Fig — P. aeruginosa ATCC 2108 (blue), E. coli ATCC 25922 (red) and K. pneumoniae ATCC 33495 (green) were co-cultured overnight, diluted, and then exposed to the specified agent for two hours. Killing was assessed visually. (left) Peptide D297 alone killed P. aeruginosa and E. coli, confirming its lack of specificity. (middle) In the absence of complement or immune cells, VSX alone had no killing effect. (right) VSX-1 demonstrated the rapid, specific, complete killing of P. aeruginosa. Little killing of E. coli was found, which confirmed the specific activity of the ADC. (TIF) [file ppat.1011612.s004.TIF]

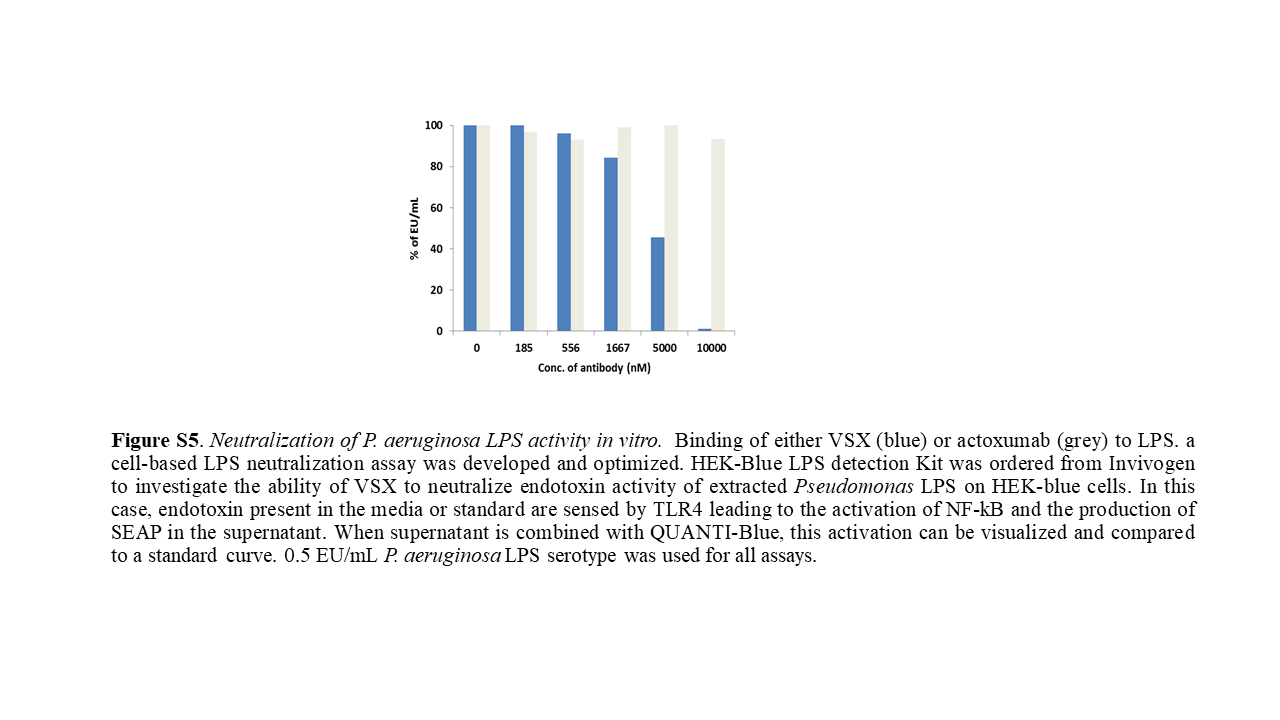

Supplement: S5 Fig — The HEK-Blue LPS detection Kit (Invivogen) was used to investigate the ability of VSX to neutralize the endotoxin activity of extracted P. aeruginosa LPS on HEK-blue cells. In this case, endotoxin present in the medium or the standard is sensed by TLR4, leading to the activation of NF-kB and the production of SEAP in the supernatant. When the supernatant is combined with QUANTI-Blue, this activation can be visualized and compared with a standard curve. 0.5 EU/ml P. aeruginosa LPS serotype was used for all assays. (TIF) [file ppat.1011612.s005.TIF]

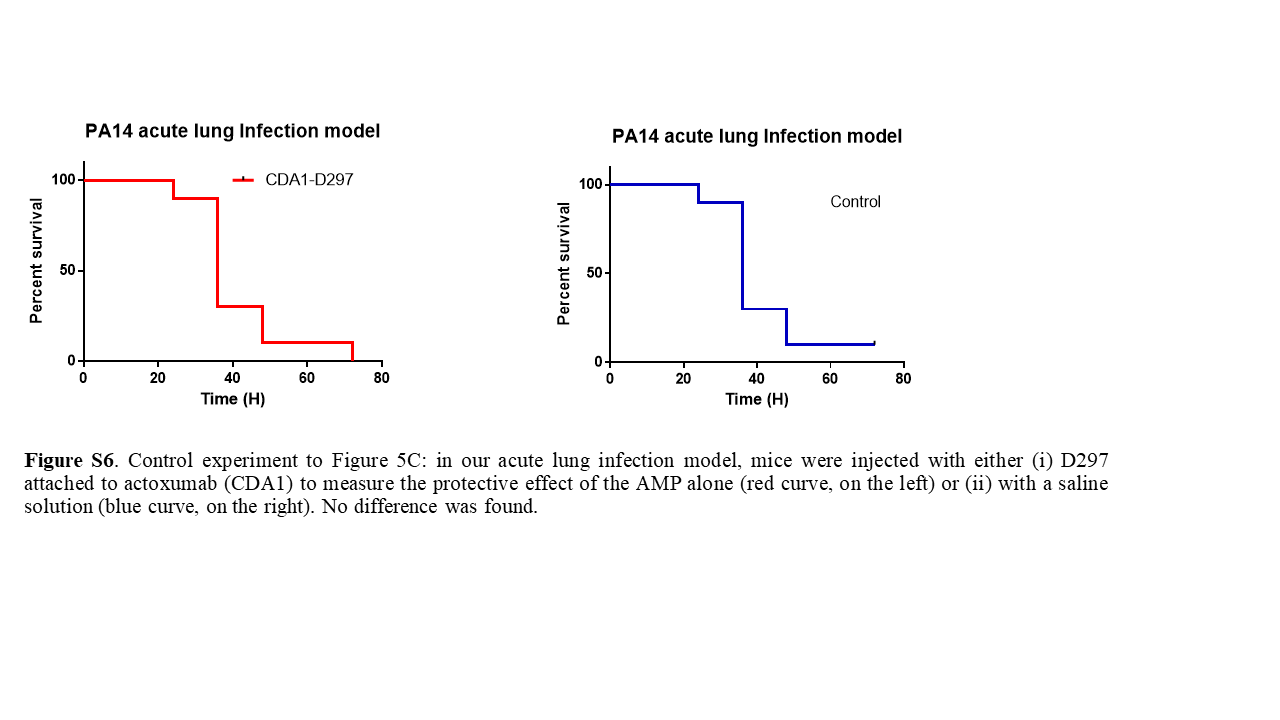

Supplement: S6 Fig — No difference was found. (TIF) [file ppat.1011612.s006.TIF]

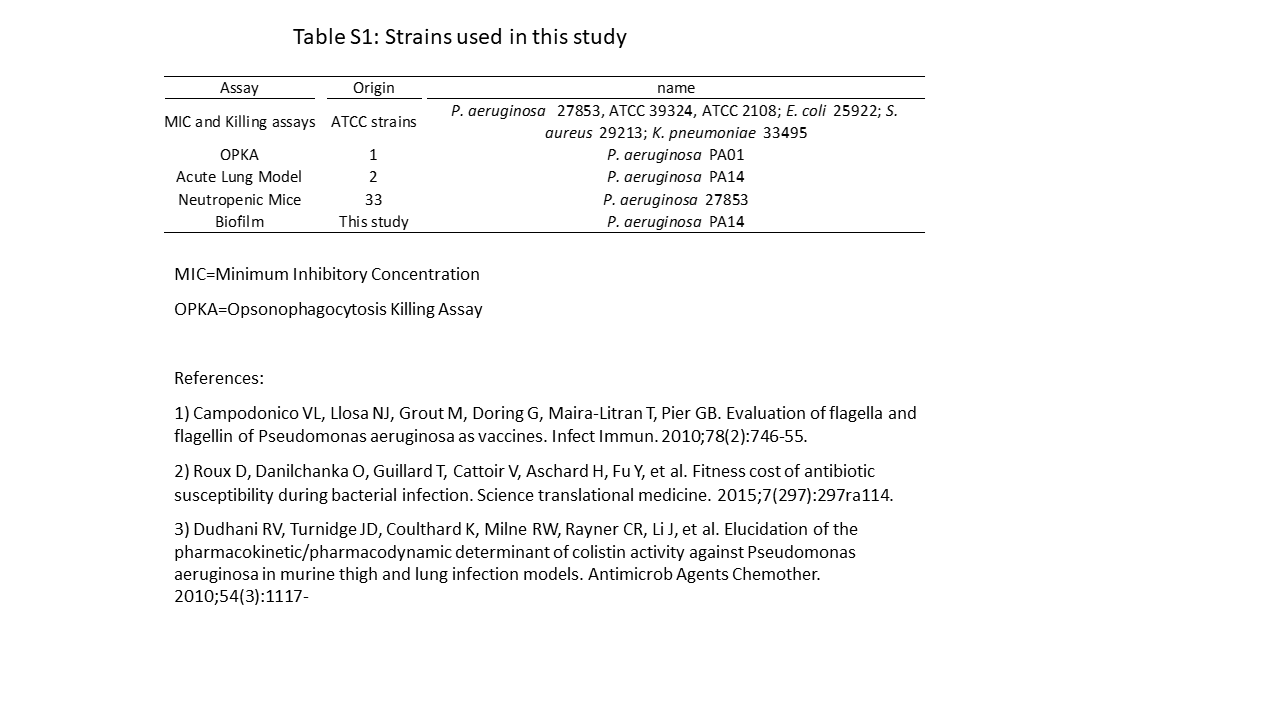

Supplement: S1 Table — (TIF) [file ppat.1011612.s007.tif]
